# Smad5 acts as an intracellular pH messenger and maintains bioenergetic homeostasis

**DOI:** 10.1038/cr.2017.85

**Published:** 2017-07-04

**Authors:** Yujiang Fang, Zhongliang Liu, Zhenyu Chen, Xiangjie Xu, Mengtao Xiao, Yanyan Yu, Yuanyuan Zhang, Xiaobai Zhang, Yanhua Du, Cizhong Jiang, Yuzheng Zhao, Yiran Wang, Beibei Fan, Daniel Terheyden-Keighley, Yang Liu, Lei Shi, Yi Hui, Xin Zhang, Bowen Zhang, Hexi Feng, Lin Ma, Quanbin Zhang, Guohua Jin, Yi Yang, Bin Xiang, Ling Liu, Xiaoqing Zhang

**Affiliations:** 1 Shanghai Tenth People's Hospital, and Neuroregeneration Key Laboratory of Shanghai Universities, Tongji University School of Medicine, Shanghai 200092, China; 2 Department of Anatomy and Neurobiology, the Jiangsu Key Laboratory of Neuroregeneration, Nantong University, Nantong, Jiangsu 226001, China; 3 The School of Life Sciences and Technology, Tongji University, Shanghai 200092, China; 4 China Novartis Institutes for BioMedical Research, Shanghai 201203, China; 5 Synthetic Biology and Biotechnology Laboratory, State Key Laboratory of Bioreactor Engineering, Shanghai Collaborative Innovation Center for Biomanufacturing Technology, East China University of Science and Technology, Shanghai 200237, China; 6 Tongji University Advanced Institute of Translational Medicine, Shanghai 200092, China; 7 The Collaborative Innovation Center for Brain Science, Tongji University, Shanghai 200092, China

**Keywords:** Smad5, intracellular pH, BMP, glycolysis, human pluripotent stem cells

## Abstract

Both environmental cues and intracellular bioenergetic states profoundly affect intracellular pH (pHi). How a cell responds to pHi changes to maintain bioenergetic homeostasis remains elusive. Here we show that Smad5, a well-characterized downstream component of bone morphogenetic protein (BMP) signaling responds to pHi changes. Cold, basic or hypertonic conditions increase pHi, which in turn dissociates protons from the charged amino acid clusters within the MH1 domain of Smad5, prompting its relocation from the nucleus to the cytoplasm. On the other hand, heat, acidic or hypotonic conditions decrease pHi, blocking the nuclear export of Smad5, and thus causing its nuclear accumulation. Active nucleocytoplasmic shuttling of Smad5 induced by environmental changes and pHi fluctuation is independent of BMP signaling, carboxyl terminus phosphorylation and Smad4. In addition, ablation of Smad5 causes chronic and irreversible dysregulation of cellular bioenergetic homeostasis and disrupted normal neural developmental processes as identified in a differentiation model of human pluripotent stem cells. Importantly, these metabolic and developmental deficits in Smad5-deficient cells could be rescued only by cytoplasmic Smad5. Cytoplasmic Smad5 physically interacts with hexokinase 1 and accelerates glycolysis. Together, our findings indicate that Smad5 acts as a pHi messenger and maintains the bioenergetic homeostasis of cells by regulating cytoplasmic metabolic machinery.

## Introduction

Given that most biological reactions are pH dependent, it is therefore not a surprise that intracellular pH (pHi) is stringently controlled within a physiological range. While changes in oxygen or nutrient supply states, extracellular pH (pHe) or osmolarity may directly affect pHi homeostasis, cells have a series of immediate pHi sensing and balancing machineries opposing these fluctuations^1,2,3,4^. To date, the pH sensitive proteins identified are exclusively membrane located transporters. The Na^+^-H^+^ exchangers and Na^+^-HCO3^−^ co-transporters are the main transporters to alkalinize cytosol, while the most common acid-importing transporters are Cl^−^-HCO3^−^ anion exchangers which acidify the cells^2^. In addition, lactate and CO_2_ generated via metabolic reactions tend to acidify the cytoplasm. Whether there is an intracellular pHi messenger which links pHi fluctuations to these metabolic reactions remains unknown.

BMP and TGFβ signaling are evolutionarily conserved pathways, which play pivotal roles in embryonic development, epithelial and mesenchymal cell states transition, and tumor metastasis^5^. Upon BMP or TGFβ stimulation, cytosolic R-Smad members, including Smad1, Smad2, Smad3, Smad5 and Smad8, become phosphorylated by the type I receptors at the most C-terminal SSXS motif^6,7^. These phosphorylated R-Smads then oligomerise with Smad4 and translocate into nucleus for gene transcriptional regulation^8^. Although amino acid sequences of R-Smads share extremely high homology, Smad1/5/8 favor BMP signaling, while Smad2/3 mainly act downstream of the TGFβ pathway^9^. Transgenic animal studies have demonstrated that Smad1/5/8 or Smad2/3 cannot fully compensate each other, although the exact mechanisms are unclear^10,11^. Recently, Smad2 has been identified as playing a non-transcriptional role in regulating mitochondrial dynamics and function. This is facilitated by complexing with Mitofusin 2, Rab and Ras interactor 1^12^, suggesting a noncanonical role of the component within the BMP and TGFβ pathways in regulating metabolic processes. In addition, Smad5 deficiency results in abnormally swollen mitochondria and apoptosis in cardiomyocytes, indicating that it might be involved in energy metabolism^13^.

Here, we show that Smad5 can actively respond to changes of pHi through nucleocytoplasmic shuttling. The MH1 domain of Smad5 plays a critical role in the pHi response, whereas the nuclear export signals (NESs) located in the linker region and the MH2 domain control the process of nuclear export. Importantly, the novel role of Smad5 in responding to pHi changes is BMP signalling independent and its nucleocytoplasmic shuttling does not require interaction with Smad4. Apart from its canonical nuclear role in transcriptional regulation, cytosolic Smad5 enhances glycolysis by interacting with hexokinase 1 (HK1). Loss of function of Smad5 causes glycolysis defects and irreversible dysregulation of cellular bioenergetics homeostasis. These data demonstrate that Smad5 is an immediate pHi fluctuation messenger and an important regulator of physiological bioenergetic homeostasis.

## Results

### Smad5 is sensitive to environmental cue fluctuations

**Figure 1:**
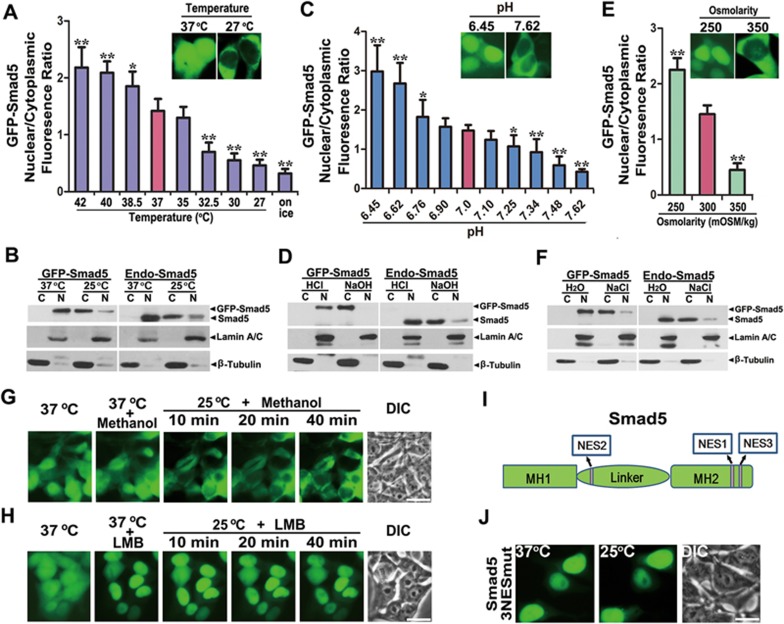
Temperature, pHe and osmolarity fluctuations dynamically control Smad5 nuclear export. **(A)** Average fluorescence quantification of nuclear and cytoplasmic localized GFP-Smad5 at various temperatures (*n* = 30; data are mean ± SEM; ^*^P < 0.05; ^**^P < 0.01). **(B)** Western blotting shows that endogenous Smad5 also undergoes temperature-sensitive cytoplasmic translocation in HEK293 cells. **(C)** Average fluorescence quantification of nuclear and cytoplasmic GFP-Smad5 at various extracellular pH values (*n* = 30; data are mean ± SEM; ^*^P < 0.05; ^**^P < 0.01). **(D)** Western blotting shows that endogenous Smad5 exhibits pHe-sensitive nucleocytoplasmic shuttling in HEK293 cells. **(E)** Average fluorescence quantification of nuclear and cytoplasmic localized GFP-Smad5 at various extracellular osmolarity (*n* = 30; data are mean ± SEM; ^*^P < 0.05; ^**^P < 0.01). **(F)** Western blotting shows that endogenous Smad5 exhibits extracellular osmolarity-sensitive nucleocytoplasmic shuttling in HEK293 cells. **(G)** Pretreatment of *GFP-Smad5*-expressing HEK293 cells with methanol for 1 h at 37 °C does not influence nucleocytoplasmic distribution of Smad5. And in the presence of methanol, low temperature triggers nucleocytoplasmic shuttling of GFP-Smad5. Scale bar, 10 μm. **(H)** 5 ng/ml LMB treatment for 1 h at 37 °C induces more obvious nuclear accumulation of GFP-Smad5 and completely blocks low temperature-triggered nucleocytoplasmic shuttling. Scale bar, 10 μm. **(I)** Schematic diagram of the positions of the three Smad5 NESs. **(J)** Mutation of all three NESs in Smad5 completely abrogates low temperature-driven Smad5 nuclear export even 1 h after shifting the cells to 25 °C. Scale bar, 10 μm.

Living organisms are imbued with the ability to sense and buffer against environmental perturbations to maintain intracellular homeostasis. Molecular mechanisms underlying the sensing of environmental stresses and maintenance of cellular homeostasis are still the focus of intense study. We unexpectedly found that GFP-Smad5 diffused from nucleus to cytoplasm within several minutes when the cells were placed at 25 °C (Figure 1A, Supplementary information, Figure S1A-S1D, Movie S1). The cytoplasmically distributed GFP-Smad5 repopulated in the nucleus 30 min after the cells were placed back at 37 °C (Supplementary information, Figure S1D). Among all Smad family proteins, Smad2, Smad4 and Smad8 are mostly distributed within the cytoplasm (Supplementary information, Figure S1E and S1F)^23^. Smad1 and Smad3 are largely distributed in the nucleus, but these proteins did not shuttle upon temperature change (Supplementary information, Figure S1D and S1G). These data strongly suggest that Smad5 differs from other Smads by virtue of its ability to sense ambient temperature fluctuation and change its nucleocytoplasmic distribution dynamically.

We then studied the temperature range that affects Smad5 nucleocytoplasmic distribution. At 37 °C, more Smad5 was found in the nucleus than in the cytoplasm (Figure 1A and Supplementary information, Figure S1H). Placing the cells at a temperature lower than 32.5 °C drove most of the nucleus-localized Smad5 to the cytoplasm. Even when the cells were directly placed on ice, Smad5 also quickly accumulated in the cytoplasm. When the environmental temperature was higher than 37 °C, more Smad5 was distributed in the nucleus (Figure 1A and Supplementary information, Figure S1H). These results suggest that Smad5 may be able to sense ambient temperature changes within a wide range. Importantly, western blotting shows that endogenous Smad5 was located largely in the nucleus at 37 °C and translocated into the cytoplasm at 25 °C, in a similar manner to that of GFP-Smad5 (Figure 1B).

The striking temperature-sensitive nucleocytoplasmic redistribution of Smad5 caused us to wonder whether other environmental changes could also induce its redistribution (Supplementary information, Figure S2A-S2C). Among these conditions, we found that changes in external pH (pHe) and osmolarity effectively regulated Smad5 nucleocytoplasmic distributions, in a similar way to that of temperature change (Supplementary information, Movie S2-S5).

GFP-Smad5 was exclusively located in the nucleus when cells were treated with 10 mM hydrochloric acid for 10 min at 37 °C (Supplementary information, Figure S3A and Movie S4). On the other hand, 2 mM sodium hydroxide rapidly induced GFP-Smad5 to translocate into the cytoplasm within 2 min at 37 °C (Supplementary information, Figure S3B and Movie S2). Statistical analysis showed that GFP-Smad5 responded to a subtle range of pHe (Figure 1C). Smad5 was mostly located in the nucleus when pHe was lower than 6.62 (Figure 1C and Supplementary information, Figure S3C), whereas with a pHe higher than 7.62, it was mostly distributed in the cytosol (Figure 1C and Supplementary information, Figure S3D). With an acidic external pH, endogenous Smad5 was again located predominately in the nucleus, whereas alkaline pHe induced its accumulation in the cytoplasm just like GFP-Smad5 (Figure 1D). Therefore, Smad5 may sense delicate pHe changes within a physiological range by nucleocytoplasmic shuttling. In addition, GFP-Smad5 showed obvious nuclear accumulation under hypotonic conditions, whereas it quickly translocated into the cytoplasm under hypertonic conditions (Figure 1E, Supplementary information, Figure S3E and S3F, Movie S3 and S5). Endogenous Smad5 also responded to fluctuations in extracellular osmolarity (Figure 1F). Moreover, these environmental cues did not affect the level of total Smad5 protein (Supplementary information, Figure S3G). Together, these data show that Smad5 is sensitive to fluctuations in temperature, pHe and osmotic pressure.

### Dynamic nuclear export of Smad5 is CRM1-dependent

Nuclear export is generally dependent on signaling and accomplished by exportins^14^. The most versatile nuclear export receptor is CRM1, which was first identified in fission yeast^15^. Leptomycin B (LMB) binds to CRM1 and inhibits the binding of RanGTP as well as other export substrates^16^. In the solvent-treated control group, methanol treatment did not cause any change in the nucleocytoplasmic shuttling of Smad5 triggered by low temperature (Figure 1G). GFP-Smad5 accumulated exclusively in the nucleus after LMB treatment at 37 °C for 1 h (Figure 1H). These data suggest that at 37 °C, GFP-Smad5 is highly dynamic and capable of both nuclear import and export. In the presence of LMB, switching the cells to 25 °C for as long as 40 min did not induce any Smad5 cytoplasmic accumulation (Figure 1H). These results indicate that low temperature-triggered Smad5 cytoplasmic accumulation is an event requiring active nuclear export and is CRM1-dependent. To further exclude the possibility that low temperature affects Smad5 nuclear import, we first placed HEK293 cells expressing *GFP-Smad5* at 25 °C for 30 min. GFP-Smad5 was found almost completely in the cytoplasm under these conditions (Supplementary information, Figure S4A and S4B). Subsequently, methanol or LMB was added to the cells at 25 °C. Strikingly, after 40 min of LMB treatment, the formerly cytoplasmic GFP-Smad5 now entirely accumulated in the nucleus at 25 °C (Supplementary information, Figure S4B). As a control, methanol treatment did not repopulate Smad5 into the nucleus at 25 °C (Supplementary information, Figure S4A). Thus, Smad5 preserves its ability to be imported into the nucleus at 25 °C but its low temperature-mediated nuclear export results in accelerated cytoplasmic accumulation of Smad5.

Classic NESs are required for CRM1-dependent nuclear export^17^. We were able to find three such NESs in the Smad5 sequence (Figure 1I). Smad5 mutants with any one of these NES sites mutated showed nuclear accumulation at 37 °C and exhibited cytoplasmic translocation at 25 °C, despite the fact that they all exhibited a slower shuttling rate compared to wild-type (WT) Smad5 (Supplementary information, Figure S4C-S4F). These data indicate that all three NESs take part in low temperature-triggered nucleocytoplasmic shuttling of Smad5 although they are not absolutely indispensable. When we mutated all three NESs in Smad5 (3NESmut), we found that the mutant Smad5 showed exclusive nuclear localization at either 37 °C or 25 °C (Figure 1J). Therefore, the nuclear export of Smad5 triggered by low temperature depends on the three NESs scattered along the linker region and the MH2 domain. In addition, the functional redundancy of the Smad5 NES elements can also account for the immediate cell response to the fluctuation of environmental cues through rapid Smad5 nuclear export.

### Nucleocytoplasmic shuttling of Smad5 in response to environmental cues is independent of BMP signaling

To date, the translocation of Smad5 from cytoplasm to nucleus has been exclusively tied to BMP signaling^18^. Interestingly, we did not detect any changes in Smad5 distribution when BMP signaling was blocked with LDN193189 (LDN) or activated with BMP4; under these conditions, low temperature persistently triggered cytoplasmic accumulation of Smad5 (Supplementary information, Figure S2A and S5A). We therefore generated a deletion mutant (GFP-Smad5 Δ11) of Smad5 by removing the C-terminal 11 amino acids that contain the BMP-responsive SSVS phosphorylation sites. We asked whether the nucleocytoplasmic shuttling of Smad5 in response to temperature is affected by this mutation. We found that GFP-Smad5 Δ11 was largely distributed in the nucleus at 37 °C, similar to WT Smad5, and translocated into the cytoplasm when cells were shifted to 25 °C (Supplementary information, Figure S5B). We then directly mutated the SSVS phosphorylation sites to AAVA (3A) or DDVD (3D) to completely block signaling-mediated phosphorylation or dephosphorylation of Smad5. Similarly, the Smad5 3A or Smad5 3D mutant showed temperature-induced nuclear export comparable to WT Smad5 (Supplementary information, Figure S5B). Even in the absence of Smad4, GFP-Smad5 shuttled from the nucleus to the cytoplasm in response to cold, alkaline pHe or hypertonic conditions, and accumulated in the nucleus under acidic pHe or hypotonic conditions (Supplementary information, Figure S5C-S5E). Collectively, these results suggest that the dynamic nucleocytoplasmic redistribution of Smad5 triggered by environmental cues is independent of BMP signaling, of carboxyl-tail phosphorylation state and of interaction with Smad4.

### The temperature, pHe and osmotic pressure response all converge to pHi

**Figure 2:**
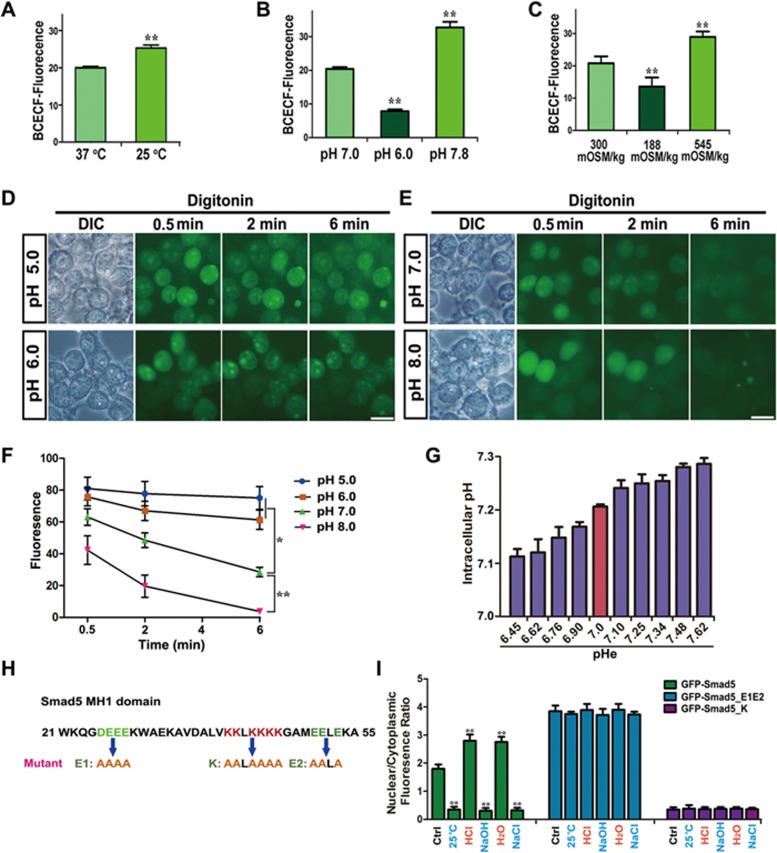
Smad5 directly senses pHi through charged amino acid clusters. **(A-C)** Quantifications of intracellular BCECF fluorescence at various temperature **(A)**, pHe **(B)** and osmolarity **(C)**. Each quantification was made from 10 independent images. Data are presented as mean ± SEM; ^**^P< 0.01. **(D**, **E)** Digitonin permeabilization experiments demonstrate that increased pH gradually promotes Smad5 nuclear export. Scale bar, 10 μm. **(F)** Average fluorescence quantification of nuclear distributed GFP-Smad5 at various pH in **D** and **E** (*n* = 30; data are presented as mean ± SEM; ^*^P < 0.05; ^**^P < 0.01). **(G)** Measurement of pHi using pHIuorin at different pHe ranges. **(H)** Diagram showing positions of acidic and basic amino acid clusters. **(I)** Quantification of nuclear and cytoplasm fluorescence ratio for GFP-Smad5, GFP-Smad5_E1E2 and GFP-Smad5_K at various conditions of temperature, pHe and osmolarity (*n* = 30; data are presented as mean ± SEM; ^**^P < 0.01).

Since nucleocytoplasmic shuttling of Smad5 responds to temperature, pHe and osmotic pressure with a very similar bidirectional pattern, we hypothesized that a common factor might link these environmental changes. Low temperature attenuates the cellular metabolic rate and decreases proton production within the mitochondria, subsequently alkalizing the cytosol. It has been demonstrated that pHe levels profoundly affect membrane transporters and lead to intracellular pH (pHi) fluctuation in the same direction^2^. High-osmotic pressure has also been shown to increase pHi^1,2^. To test whether temperature, pHe and osmotic pressure indeed converge on pHi, we measured pHi using BCECF-AM^19^. BCECF fluorescence intensity increased in HEK293 cells shifted from 37 °C to 25 °C, indicating that low temperature induces cytosolic alkalization (Figure 2A and Supplementary information, Figure S6A). The addition of hydrochloric acid (10 mM) caused intracellular acidification and decreased BCECF fluorescence intensity whereas potassium hydroxide (2 mM) treatment rapidly increased pHi (Figure 2B and Supplementary information, Figure S6B). A hypotonic environment also triggered cytosol acidification, whereas hypertonic conditions led to a pHi increase (Figure 2C and Supplementary information, Figure S6C). Taken together, our data confirmed that the response to fluctuation in temperature, pHe and osmotic pressure all converged on a change in pHi.

To further verify that pHi directly regulates Smad5 nucleocytoplasmic shuttling, we treated *GFP-Smad5* HEK293 cells with the ionophoric uncoupler of oxidative phosphorylation, FCCP, for 30 min to decrease pHi; this resulted in nuclear accumulation of Smad5 (Supplementary information, Figure S6D and S6E). In addition, we pretreated HEK293 cells carrying the *GFP-Smad5* transgene with 10 mM hydrochloric acid for 10 min to trigger nuclear accumulation of Smad5. Cells were then permeabilized by treatment with 160 μg/ml digitonin for 1 min, after which they were soaked in solutions of pH 5.0, 6.0, 7.0 or 8.0. At pH 5.0 or 6.0, nuclear export of Smad5 was almost completely repressed (Figure 2D and 2F). In contrast, GFP-Smad5 was able to exhibit nuclear export at pH 7.0 or 8.0 (Figure 2E and 2F). To determine whether Smad5 responds to pHi changes within a normal physiological range, we measured pHi using pHIuorin^20,21^ at different pHe as shown in Figure 1C. Statistical analysis showed that pHi increased by 0.17 units when the corresponding pHe changed from 6.45 to 7.62 (Figure 2G), indicating that Smad5 could respond to a subtle physiological range of pHi changes by nucleocytoplasmic shuttling. These data strongly suggest that pHi is a potent factor that regulates the nuclear export rate of Smad5, thus contributing to its dynamic nucleocytoplasmic distribution that is influenced by fluctuations in extracellular cues.

### The MH1 domain of Smad5 is essential for its pHi response

Next, we wondered which subdomain of Smad5 responds to pHi changes. To this end we constructed a series of chimeric molecules between Smad1 and Smad5, which are highly homologous but show completely different nucleocytoplasmic translocation properties (Supplementary information, Figure S6F-S6I). Interchanging the MH2 domains between Smad1 and Smad5 did not significantly affect their subcellular distribution at either 37 °C or 25 °C (Supplementary information, Figure S6G). Smad5 maintained its specific properties and could translocate into the cytoplasm at 25 °C upon removal of its entire MH2 domain (Supplementary information, Figure S6H). We then interchanged the MH1 domains of Smad1 and Smad5 and studied their distribution at 37 °C or 25 °C. Interestingly, GFP-Smad5 harboring the MH1 domain of Smad1 behaved similarly to WT GFP-Smad1, and resided in the nucleus at both 37 °C and 25 °C (Supplementary information, Figure S6I). However, GFP-Smad1 harboring the MH1 domain of Smad5 behaved like Smad5 and exhibited nuclear localization at 37 °C but shuttled into the cytoplasm within 10 min at 25 °C (Supplementary information, Figure S6I). Therefore, we conclude that the MH1 domain of Smad5 plays a critical role in responding to pHi changes, while the linker region and MH2 domain contribute to nuclear export at alkaline pHi.

We next asked whether Smad5 directly responds to pHi changes and, if so, what would be the amino acid residues in the MH1 domain responsible for such a role. Because this is a response to pH, we first sought to identify acidic or basic amino acid residues within the MH1 domain that may be essential for the Smad5 pHi response. We identified two acidic and one basic clusters of such charged amino acids in the Smad5 MH1 domain and mutated these residues to alanines (Figure 2H). Compared to the WT, the combined mutant of Smad5's two acidic domains_E1, E2 (E1E2) showed biased nuclear accumulation at 37 °C and exhibited attenuated nuclear export capacity under cold, alkaline pHe or hypertonic conditions (Figure 2I and Supplementary information, Figure S6J). Conversely, the basic domain mutant, Smad5_K, showed cytoplasmic accumulation under normal culture conditions, and acidic pHe or hypotonic environments now failed to promote its nuclear accumulation (Figure 2I and Supplementary information, Figure S6J). Taken together, these data strongly suggest that these three charged amino acid clusters in the MH1 domain of Smad5 are required for the direct sensing of pHi.

### *Smad5* deficiency causes downregulation of metabolism-related genes

To investigate the biological functions of this highly dynamic and pHi-sensitive protein, we deleted *Smad5* in human embryonic stem cells (hESCs) through donor-mediated homologous recombination in combination with transcription activator-like effector nucleases (TALENs)-induced DNA cleavage (Supplementary information, Figure S7A)^22,23^. Knockout (KO) success was confirmed by genomic PCR and western blotting (Supplementary information, Figure S7B and S7C). A similar strategy was applied to generate HEK293 and HeLa *Smad5* KO cell lines (Supplementary information, Figure S7D and S7E).

**Figure 3:**
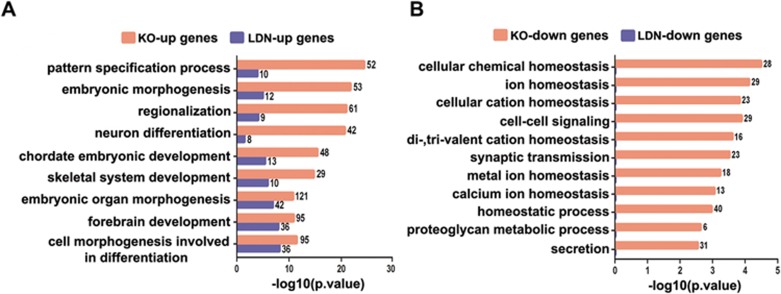
*Smad5* KO induces specific transcriptional profiling changes related to metabolic homeostasis. **(A**, **B)** Functional annotation of up or downregulated gene categories in *Smad5* KO and LDN193189-treated hES cells identified by GO.

RNA-seq was performed to systematically compare the transcriptomes of WT, *Smad5* KO and LDN-treated hES cells. Data retrieved from three independent WT and KO lines revealed ∼468 upregulated and ∼438 downregulated genes upon *Smad5* KO (Supplementary information, Figure S7F and S7G). There was a significant overlap between up and downregulated genes among these three WT and KO lines (Supplementary information, Figure S7H). We also treated hES cells with LDN, which prevented Smad1/5/8 SSXS phosphorylation (Supplementary information, Figure S8A) and then analyzed the transcriptome profiles. This showed that LDN treatment induced much fewer changes in gene expression compared to the *Smad5* KO (Supplementary information, Figure S8B). Among those upregulated or downregulated genes, only 32 or 1, respectively, overlapped between the *Smad5* KO and LDN groups (Supplementary information, Figure S8C). This suggests that disrupting *Smad5* results in a different gene expression profile compared to the blocking of BMP signaling. Gene ontology (GO) analyses showed that upregulated genes in the *Smad5* KO group were mainly involved in the biological processes of embryonic development and patterning and showing a certain degree of similarity with LDN upregulated genes (Figure 3A). Downregulated gene groups in *Smad5* KO cells belonged mainly to the category of cellular hemostasis including cellular chemical homeostasis; ion homeostasis; and proteoglycan metabolic processing. These functions have no overlap with those of LDN downregulated genes (Figure 3B). The expression patterns of the top 10 most downregulated genes were also validated by qPCR in WT, *Smad5* KO, LDN-treated and *Smad4* KO cells (Supplementary information, Figure S8D).

We then reintroduced *Smad5* or *GFP-Smad5* into the *Smad5* KO hES cell line through lentiviral transduction (Supplementary information, Figure S9A). Surprisingly, neither *Smad5* nor *GFP-Smad5* restored the expression of those downregulated genes caused by disruption of endogenous *Smad5* (Supplementary information, Figure S9B). We therefore carried out pre-knockout rescue, in which *Smad5* or corresponding mutants were first overexpressed and then followed by endogenous *Smad5* ablation. KO or overexpression efficacy was verified by genomic PCR and western blotting (Supplementary information, Figure S9C and S9D). Under these conditions, downregulation of gene expression induced by the *Smad5* KO was significantly rescued by pre-expression of *Smad5* (Supplementary information, Figure S9E).

We found that Smad5_E1E2 was mostly present in the nucleus, whereas GFP-Smad5_K was largely distributed in the cytoplasm (Figure 2I and Supplementary information, Figure S6J). A co-immunoprecipitation (Co-IP) experiment showed that BMP4 stimulation significantly triggered the interaction of WT Smad5 with Smad4. In contrast, GFP-Smad5_K showed no significant Smad4 interaction (Supplementary information, Figure S9F). Notably, pre-rescue with *Smad5_K*, but not *Smad5_E1E2*, largely prevented *Smad5* KO-induced downregulation of gene expression (Supplementary information, Figure S9E). Genome sequencing confirmed the genomic DNA integrity of the transduced *Smad5_E1E2* after TALENs targeting (Supplementary information, Figure S9G). The differences between Smad5_K and Smad5_E1E2 in rescuing expression of genes resulting from the *Smad5* KO further indicate that it is cytoplasmic Smad5 but not nuclear Smad5 or the BMP/Smad5 signaling pathway, that maintains a normal cellular homeostatic state. Moreover, acidic and basic pH treatment showed that the shuttling of Smad5 into or out of the nucleus for various time periods could not acutely regulate expression of the above characteristic genes (Supplementary information, Figure S10A-S10C), thus indicating a non-transcriptional role of Smad5 in this process.

### Disruption of *Smad5* reduces glycolytic rate

**Figure 4:**
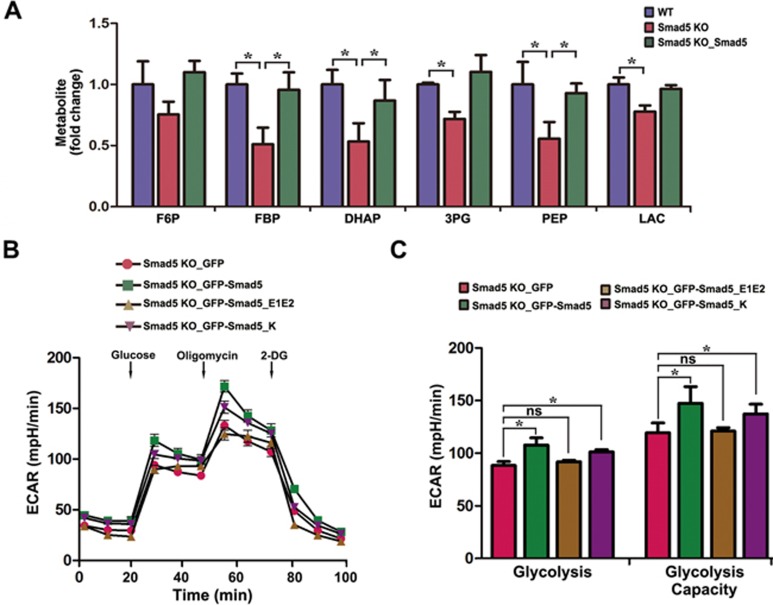
Loss of function of Smad5 causes glycolysis defects. **(A)** LC-MS/MS analysis of abundance in glycolytic intermediates in WT, *Smad5* KO and *Smad5* pre-rescue hES cells. Data are represented as mean ± SEM of three independent experiments. Unpaired two-tailed Student's *t*-test. ^*^P < 0.05. **(B)** *GFP*, *GFP-Smad5*, *GFP-Smad5_E1E2* and *GFP-Smad5_K* were expressed in *Smad5* KO HEK293 cells, and extracellular acidification rate (ECAR) was measured with the Seahorse Analyzer (*n* = 6). **(C)** Statistics of glycolysis and glycolysis capacity in **B**. Data are represented as mean ± SEM of six independent experiments. Unpaired two-tailed Student's *t*-test. ^*^P < 0.05.

To further delineate the cellular homeostatic deficits in *Smad5* KO cells, we profiled metabolites in WT, *Smad5* KO and *Smad5* KO with *Smad5* pre-expression hES cell lines using liquid chromatography tandem mass spectrometry (LC-MS/MS). The *Smad5* deficiency resulted in lower levels of glycolytic intermediates. These levels were largely rescued by pre-expression of *Smad5* (Figure 4A), indicating that Smad5 is required for maintaining a normal glycolysis rate. In addition, the extracellular acidification rate (ECAR) exhibited that glycolysis was rescued by GFP-Smad5 and GFP-Smad5_K, but not GFP-Smad5_E1E2, suggesting that cytoplasmic Smad5 can promote a higher glycolysis rate (Figure 4B and 4C). In contrast, Smad1 and Smad8 did not affect the glycolysis rate (Supplementary information, Figure S11A and S11B). Together, these results suggest that Smad5 is an important regulator of the rate of glycolysis.

### *Smad5* KO induces irreversible cellular bioenergetic homeostasis and impairs mitochondrial respiration

**Figure 5:**
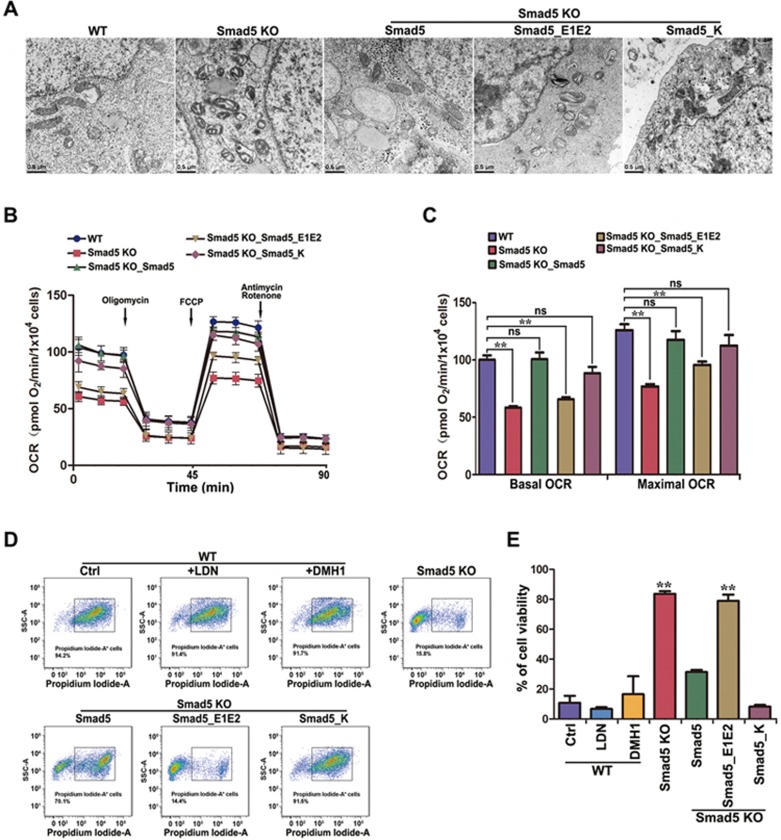
*Smad5* KO causes deficiency in mitochondrial respiration and malfunction of cellular bioenergetic homeostasis. **(A)** Electron microscopy images showing mitochondrial morphology in WT, *Smad5* KO and pre-rescue hES. Scale bar, 0.5 μm. **(B)** Oxygen consumption rate (OCR) changes under mitochondrial stress in WT, *Smad5* KO and pre-rescue hES as measured using the Seahorse Analyzer (*n* = 6). **(C)** Statistics of basal and maximal OCR in **B**. Data are represented as mean ± SEM of 6 independent experiments. Unpaired two-tailed Student's *t*-test. ^**^P < 0.01. **(D)** Flow cytometry sorting of hES cells after propidium iodide staining. **(E)** Statistics of cell viability following serum withdrawal. Data are presented as mean ± SEM in three independent experiments. ^**^P < 0.01.

Mitochondria play a central role in cellular respiration and energy production^24,25^. Transmission electron microscopy showed that the mitochondria of *Smad5* KO hES cells mostly exhibited swollen and vacuolated features (Figure 5A), suggesting severe mitochondrial dysfunction. Re-expressing *Smad5* in *Smad5* KO hES cells through lentiviral transduction could not rescue the abnormal mitochondrial morphology, suggesting that the mitochondrial dysfunction caused by *Smad5* KO is irreversible (Supplementary information, Figure S12). The abnormal mitochondrial morphology in *Smad5* KO cells can, as predicted, be rescued by pre-expression of ectopic *Smad5* or *Smad5_K*, but not *Smad5_E1E2* (Figure 5A), further suggesting a cytoplasmic role of Smad5 in maintaining normal mitochondrial function. We next used a flux analyzer to evaluate the oxygen consumption rate (OCR). Both the basal OCR and maximal OCR decreased in *Smad5* KO hES cell lines (Figure 5B and 5C) and this could be rescued by pre-expression of *Smad5* and *Smad5_K*, but not *Smad5_E1E2*. We also found the same phenomenon in HEK293 cells and HeLa cells (Supplementary information, Figure S13A-S13D). In striking contrast to the *Smad5* KO, loss of *Smad4* did not profoundly affect the maximal OCR (Supplementary information, Figure S13E and S13F). Although re-introducing *GFP-Smad5* into *Smad5* KO HEK293 cells increased OCR, overexpression of *GFP-Smad1* and *GFP-Smad8* had no effect on either basal or maximal OCR (Supplementary information, Figure S13G and S13H).

Furthermore, considering the obligatory role of Smad5 in maintaining bioenergetic homeostasis, we wondered whether *Smad5* KO cells could tolerate acute nutrient stress such as serum deprivation. Surprisingly, we found that WT hES cells were sensitive to nutrient stress and could hardly survive upon removal of serum from the culture. Propidium iodide-A staining followed by flow cytometry analysis revealed massive amounts of cell death 3 days after serum deprivation (Figure 5D and 5E). Remarkably, *Smad5* KO hES cells survived well under these extreme nutrient stress conditions (Figure 5D and 5E), suggesting a completely transformed bioenergetic homeostasis state. It should be noted that this abnormal bioenergetic homeostasis state of *Smad5* KO cells is not related to deficiencies in BMP signaling, since neither LDN nor DMH1 pretreatment of WT cells could recapitulate the improved survival of *Smad5* KO cells under serum deprivation conditions (Figure 5D and 5E). Moreover, pre-expression of ectopic *Smad5* or *Smad5_K*, but not *Smad5_E1E2*, successfully prevented the transformation of bioenergetic homeostasis (Figure 5D and 5E).

### *Smad5* KO disrupts neural differentiation of hES cells

**Figure 6:**
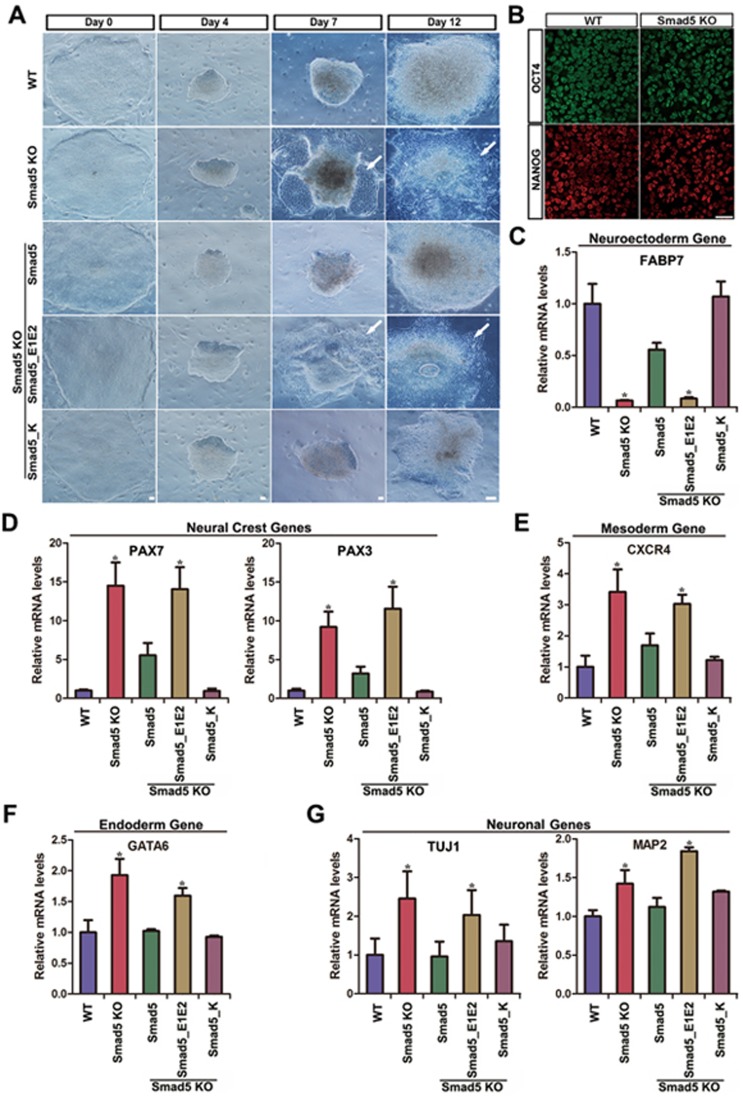
*Smad5* KO hES impairs normal neural development processes. **(A)** Bright field images show distinct steps of neural induction in WT, *Smad5* KO and pre-rescue hES. Arrowhead shows the abnormal neural morphology. Day 0, hES stage; Day 4-7, neuroectoderm; Day 12, neural progenitors. Scale bars, 25 μm. **(B)** *Smad5* KO had no effect on hES pluripotency by criterion of OCT4 and Nanog immunofluorescence staining at Day 0. Scale bar, 25 μm. **(C-H)** The relative mRNA expression level of neuroectoderm, neural crest, mesoderm, endoderm and neuronal genes in neural progenitor stage at day 12. Data are presented as mean ± SEM of three independent experiments. ^*^P < 0.05.

Neural specification is a crucial developmental process which is tightly linked to both BMP signaling and metabolic processes^26^. Increased pHi is required for embryonic stem cell differentiation^27^, and more Smad5 was located in the cytosol during early neural differentiation (Supplementary information, Figure S14A and 14B). We therefore explored the function of Smad5 in a neural differentiation model^26,28^. *Smad5* KO hES cells could maintain an ESC-like morphology, and pluripotency transcription factors were equally expressed in WT and *Smad5* KO cells (Figure 6A and 6B). However, *Smad5* KO cells differentiated toward a neural lineage in an unsynchronized fashion compared to WT cells at day 7 or day 12 (Figure 6A). The abnormal neural morphology in *Smad5* KO cells could be rescued by pre-expression of *Smad5* and *Smad5_K*, but not *Smad5_E1E2* (Figure 6A). In accord with these morphological findings, qPCR analysis of *Smad5* KO cells at day 12 showed lower expression of neuroectodermal genes, but mesendodermal genes, neural crest genes and neuron maturation genes were expressed at higher levels. This further suggests a disrupted neural developmental process (Figure 6C-6G). Again, these abnormal gene expression patterns could be pre-rescued by *Smad5* or *Smad5_K*, but not *Smad5_E1E2* (Figure 6C-6G). Together, these results suggest that cytoplasmic Smad5 possesses an important role in guiding neural development and that the bioenergetic state maintained by cytoplasmic Smad5 might be required for normal neural development.

### Smad5 maintains bioenergetic homeostasis by interacting with HK1

**Figure 7:**
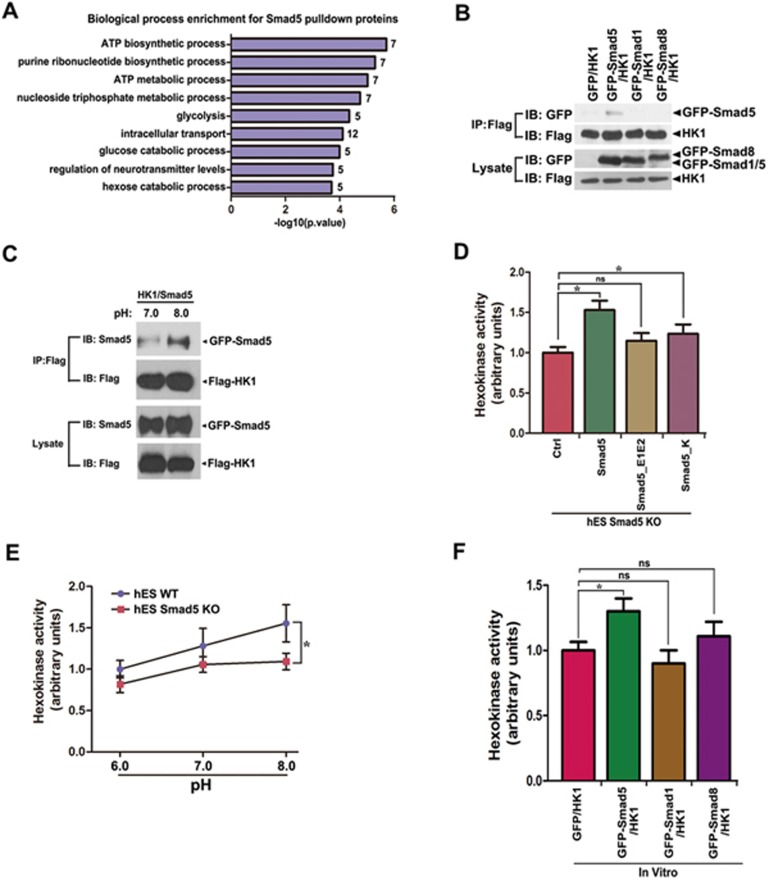
Smad5 dynamically regulates HK1 activity. **(A)** GO analysis of 67 potential Smad5 binding proteins identified by IP/MS. **(B)** HEK293 cells co-expressing of *Flag-HK1* and *GFP*, *GFP-Smad5*, *GFP-Smad1*, *GFP-Smad8* were immunoprecipitated with the GFP antibody, and specific interaction of GFP-Smad5 with Flag-HK1 is shown by western blotting. **(C)** HEK293 cells were cotransfected with *GFP-Smad5* and *Flag-HK1*. Extracellular pH was then adjusted to 7.0 or 8.0 for 30 min followed by immunoprecipitation with the anti-Flag antibody. A larger amount of Smad5 interacts with HK1 at pHe 8.0 as compared with that of pHe 7.0. **(D)** Hexokinase enzyme activity was detected in *Smad5* KO and various pre-rescue hES. Data are presented as mean ± SEM of three independent experiments. ^*^P < 0.05. **(E)** WT and *Smad5* KO hES cells were incubated in pH 6.0, pH 7.0 and pH 8.0 buffer for 30 min, respectively, followed by hexokinase enzyme activity detection. Data are presented as mean ± SEM of three independent experiments. ^*^P < 0.05. **(F)** GFP-Smad5 binding leads to HK1 activity increase *in vitro*. HK1 activity is measured *in vitro* after incubation of GFP, GFP-Smad5, GFP-Smad1 or GFP-Smad8 proteins using hexokinase enzyme activity assay kit. Data are presented as mean ± SEM of three independent experiments. ^*^P < 0.05.

To dissect the underlying molecular mechanisms by which pHi-sensitive Smad5 maintains bioenergetic homeostasis, we performed Co-IP followed by mass spectrometry (IP/MS) to investigate the Smad5 interactome. We identified 67 potential binding partners of GFP-Smad5 (Supplementary information, Table S1). GO analysis of these candidates showed that they were mainly involved in ATP biosynthetic and metabolic processes (Figure 7A), supporting the above conclusion that Smad5 plays an important role in maintaining bioenergetic homeostasis in the cytoplasm. Among these candidates, we identified HK1, a rate-limiting enzyme of glycolysis^29,30,31,32^. Ectopic expression of *GFP-Smad5* and *Flag-HK1* in HEK293 cells confirmed their interaction (Figure 7B, Supplementary information, Figure S15A and S15B). Smad1 and Smad8 did not interact with HK1 (Figure 7B). More importantly, when pHe was adjusted from 7.0 to 8.0, the interaction between Smad5 and HK1 was greatly potentiated, coinciding with the cytoplasmic accumulation of Smad5 under the alkaline pHe conditions (Figure 7C).

*In vitro* analysis revealed that HK enzymatic activity decreased in hES, HEK 293 and HeLa cells when the *Smad5* gene was deleted (Supplementary information, Figure S15C). HK activity could be pre-rescued by *Smad5* or *Smad5_K*, but not *Smad5_E1E2* in *Smad5* KO hES or HEK293 cells (Figure 7D and Supplementary information, Figure S15D). Moreover, HK activity gradually increased when pHe was shifted from 6.0 to 8.0 in WT hES, HEK293 and HeLa cells (Figure 7E, Supplementary information, Figure S15E and S15F). However, this tendency of HK activity to increase in response to pHe alkalization was significantly abrogated in *Smad5* KO cells (Figure 7E, Supplementary information, Figure S15E and S15F).

We then re-constructed HK1 enzymatic activity *in vitro* by purifying Flag-HK1 from *Smad5* KO HEK293 cells. Strikingly, supplying GFP-Smad5, but not GFP, GFP-Smad1 or GFP-Smad8, led to a significant increase in HK1 activity (Figure 7F), demonstrating a direct role of GFP-Smad5 in HK1 activation. Together, these data suggest that during pHi fluctuation, cytoplasmically localized Smad5 interacts with HK1, thus increasing HK1's enzymatic activity as a glycolytic regulator.

## Discussion

Smad5, together with Smad1 and Smad8, act as important substrates for BMP receptors, which play critical roles in embryogenesis and tissue homeostasis^33,34^. Smad1/5/8 are closely related and share similar sequences. However, our present results show that Smad5 differs from Smad1/8 in several aspects. First, these molecules have different subcellular locations. Smad5 is located both in the cytoplasm and nucleus; Smad1 is largely distributed in the nucleus; and Smad8 is mostly cytoplasmic. Differential subcellular protein localization suggests that their functions may be different. Second, the results presented here demonstrate that Smad5 is sensitive to pHi changes. Basic pHi promotes Smad5 to translocate from nucleus to cytoplasm, whereas it accumulates in the nucleus when pHi decreases. In contrast, Smad1 and Smad8 do not shuttle upon pHi fluctuations. Notably, the charged amino acid clusters in the Smad5 MH1 domain that respond to pHi changes are also found in Smad1 and Smad8, indicating that these amino acid clusters are necessary but not sufficient for the translocation of Smad5. In addition, Smad5 can act in a transcription-independent manner to regulate glycolysis and mitochondrial respiration, thus maintaining cellular bioenergetic states, while Smad1 and Smad8 cannot. Together, these results show that Smad5 possesses unique roles that differ from those of Smad1 and Smad8.

Intracellular pH plays an important role in maintaining the structure and function of proteins, regulating intracellular metabolic reactions and protein interactions. Hence pHi is stringently regulated^35^. Our findings demonstrate that Smad5 actively senses pHi through nucleocytoplasmic shuttling. Importantly, the pHi changes sensed by Smad5 are within a physiologically relevant range. We show that Smad5 can sense pHi fluctuations and subsequently regulate intracellular metabolic reactions. Therefore, Smad5 plays a key role in pHi sensing and modulation. pHi dynamics also play an essential role in development: increasing pHi is necessary for mouse embryonic stem cell (mES) differentiation, while lower pHi impairs mES differentiation^27^. Our data also show that pHi increases during neural specification, and that *Smad5* KO impairs the differentiation of hES cells towards a neural lineage. Taken together, our findings underscore the importance of Smad5 in pHi sensing and development.

The specific subcellular localization of a protein is crucial for its normal function^36,37,38^. Transcription factors, protein/RNA chaperones, cell cycle modifiers and various signaling components show precise regulation of their subcellular distribution^38,39,40,41^. Usually, translocation of a protein is regulated by signaling-mediated phosphorylation/dephosphorylation, protein-protein interactions and ATP/ADP or GTP/GDP binding states^8,38,39,40,41^. Here we demonstrate that Smad5 is a highly dynamic protein that frequently translocates between the nucleus and the cytoplasm, providing the first example of a protein whose subcellular localization can be dramatically regulated by temperature, pHe, osmolarity as well as pHi. pHi is fundamental for basic cellular biochemistry including enzyme activity, energy metabolism, cell proliferation and migration^42,43^. Empirically, pHi can be monitored by pH-sensitive dyes, microelectrodes or MRI^44^. Since Smad5 is highly pHi sensitive, it may also be applied to dynamically monitor pHi within the physiological range in *in vitro*-cultured cells or in living animals.

In conclusion, our current work demonstrates a noncanonical role of Smad5 in responding to pHi changes and regulating cellular bioenergetic states by controlling the rate of glycolysis. The role of Smad5 in regulating glycolysis by interacting with HK1 may be direct, because the enzymatic activity of HK1 reconstituted *in vitro* could be modified by adding purified Smad5 and second, the glycolysis rate in *Smad5* KO hES cells could be rescued by either post-knockout or pre-knockout re-expression of *Smad5* (Supplementary information, Figure S15G and S15H). However, it is also noteworthy that disruption of Smad5 induces an irreversible cellular bioenergetic homeostasis transformation, as evidenced by permanent mitochondrial dysfunction, which could not be reversed by post-knockout re-expression of *Smad5* (Supplementary information, Figure S12A). Therefore, Smad5 may play multiple roles in both the immediate balancing and long-term maintenance of cellular bioenergetic states through sensing environmental stresses by acting as a pHi messenger.

## Materials and Methods

### Plasmids construction

*GFP-Smad5*, *Smad5* and its mutants were cloned into the pLVX-Tight-Puro vector (Clontech). The pLVX-ef1a-rtTA advanced lentiviral vector was used together with the pLVX-Tight-Puro expression vector for making inducible overexpression lines.

### Cell culture and generation of stable cell lines

Human Embryonic Kidney (HEK293FT, Invitrogen) cells were regularly cultured in Dulbecco's Modified Eagle's Medium supplemented with 10% fetal bovine serum (GIBCO) in 5% CO_2_ at 37 °C. Human ES cells (H9, WiCell) were cultured on irradiated mouse embryonic fibroblasts (MEF) as previously described^45^. Lentiviruses were packaged in HEK293FT cells as previously described^45^. Viruses were then concentrated by ultracentrifugation and used for making stable lines. Mycoplasma detection tests were conducted in a weekly base to ensure exclusion of any contaminated cells.

### Immunofluorescence

HEK293 cell lines stably infected with *GFP* or *GFP-Smad* genes were grown on coverslips in 24-well plates. For studying protein distribution at 37 °C, cells were treated with doxycycline for 24 h to induce protein expression and were then immediately fixed for 10 min at 37 °C in 4% paraformaldehyde (PFA)/PBS pre-warmed to 37 °C. Nuclei were stained with Hoechst for 5 min after membrane permeabilization with 0.2% Triton X-100/PBS. To study protein distribution at 25 °C, cells were moved to a 25 °C incubator supplied with 5% CO_2_ for the indicated time intervals and then fixed for 10 min at 25 °C with 25 °C pre-conditioned 4% PFA/PBS. To study protein distribution at various pH or osmotic pressures, cells were treated with hydrochloric acid, sodium hydroxide, pure water or sodium chloride for 10 min at 37 °C, and then fixed for 10 min at 37 °C with 4% PFA/PBS pre-warmed to 37 °C.

### Nuclear and cytoplasmic fractionation and western blotting

HEK293 cells growing in 10 cm dish for 48 h were given indicated treatments for 15 min including temperature shifts (37 °C and 25 °C); extracellular pH changes (10 mM HCl and 2 mM NaOH); or changes in external osmotic pressure (250 mOSM/kg or 350 mOSM/kg adjusted through H_2_O or NaCl). Cells were then permeabilized using 3 ml of a physiologically isotonic solution (140 mM NaCl, 5 mM KCl, 1 mM MgCl_2_, 1 mM CaCl_2_, 10 mM glucose and 10 mM MOPS (pH 6.0 for conditions: 37 °C, extracellular pH 6.0, osmotic pressure 250 mOsm/kg; pH 7.2 for conditions: 25 °C, extracellular pH 7.6, osmotic pressure 350 mOsm/kg)) containing 160 μg/ml digitonin (Sigma) for 3 min. Nuclei were pelleted by centrifugation at 300× *g* for 5 min and supernatants containing cytoplasmic proteins were collected. Western blotting was performed following electrophoresis on a 10% SDS-polyacrylamide gel. The antibodies used for immunoblotting were rabbit anti-Smad5 (1:1 000, Cell Signaling, 9517), mouse anti-Lamin A/C (1:500, Santa Cruz, SC-7292), mouse anti-β-Tubulin (1:4 000, Sigma, T5201).

### Measurement of pHi

The pHi was measured using the pH-sensitive fluorescent probe BCECF-AM [2′,7′-bis-(2-carboxyethyl)-5-carboxyfluorescein-acetoxymethyl ester; Beyotime, China]^19^. HEK293 cells were incubated for 20 min at 37 °C with 1 μM BCECF-AM in Hank's balanced salt solution. Cells were then rinsed twice for 5 min intervals to fully remove the dye and were treated under differing conditions of: temperature (37 °C and 25 °C); extracellular pH (pH 6.0, pH 7.0, pH 7.8); and external osmotic pressure (188 mOsm/kg, 300 mOsm/kg, 545 mOsm/kg). BCECF fluorescence was imaged using a standard fluorescence filter set^19,46^.

The cytosolic pH in live cells was measured using pHIuorin^20,21^, a genetically encoded pH indicator. Cells were seeded in black 96-well flat bottom plates, transfected with the plasmid DNA to express *pHIuorin* for 30 h. Cells were washed twice with PBS in each well after carefully removing the medium. Readings were recorded after adding 100 μl PBS for 30 min at 37 °C by a Synergy 2 Multi-Mode Microplate Reader (BioTek) with excitation filters of 400 BP 10 nm and 485 BP 20 nm, and emission filter of 528 BP 20 nm (for both excitation wavelengths). Fluorescence values were corrected for background fluorescence by subtracting the intensity of the cell samples not expressing sensors. All samples were run in triplicate.

### Derivation of *Smad5* and *Smad4* knockout cell lines

The *Smad5*-targeting donor vector comprised a 766 bp 5′ homology arm, puro resistance gene and a 764 bp 3′ homology arm. TALENs were designed and assembled as described^47,48^. *Smad5*-targeting sequences were TGTCAAATGACGTCAATGGC for left TALEN, CTAGTCCAGCAGTAAAGCGA for right TALEN. TALEN-mediated homologous recombination in hES cells was performed as previously described^48^. Genomic DNA PCR primers are as follows: HR-F: AGCATATGGCACTTGTGAAG, Puro-F: TTCCTGGCCACCGTCGGCGTCTC, HR-R: CTTTAGCTCATGATGACTCTGC. Construction of the *Smad4* knockout HEK293 cell lines was described as previously^23^.

### Co-immunoprecipitation and western blotting

For co-immunoprecipitation analysis, cells were lysed in IP buffer (50 mM Tris-HCl pH 7.4, 150 mM NaCl, 1 mM EDTA, 1 mM NaF, 1% Triton X-100, 0.25% sodium deoxycholate and 5% glycerol) supplemented with protease inhibitors and phosphatase inhibitors. Total lysates were incubated in the presence of 1 μg primary GFP antibody (A6455, life technologies) mixed with 30 μl of 50% slurry Protein-G Sepharose beads (Roche) or incubated with anti-Flag M2 beads (Sigma) according to the manufacturer's protocols. Immunoprecipitates and total lysates were subjected to immunoblotting with the indicated antibodies.

### Metabolism analysis

Targeted LC-MS/MS-based metabolomics analysis was performed as previously described^49^. 10^7^ hES cells were harvested in 80% (v/v) methanol at −80 °C. Insoluble material in lysates was centrifuged at 12 000 rpm for 15 min, and the resulting supernatant was evaporated using a refrigerated speed vac.

### Seahorse assay

hES cells were cultured on feeder cells in XF96 cell culture microplate (Seahorse Bioscience). HEK293 and Hela cells were seeded onto a Matrigel-coated XF96 cell culture microplate. ECAR and OCR were measured using a standard protocol (Seahorse Bioscience) and were normalized using counted cell numbers.

### Electron microscopy

hES cells were fixed overnight at 4 °C in phosphate buffer (PB) containing 2% glutaraldehyde. After five washes in PB buffer (pH 7.4) for 5 min each, cells were post-fixed for 1 h at RT in 1% osmium tetroxide (OSO_4_). After further washes with PB, cells were dehydrated in 30%, 50%, 70% and 90% acetone (30 min per step), and the cells were then further dehydrated in 100% acetone three times for 11 min each at RT. The samples were then infiltrated in 3:7 (vol/vol) acetone/epon for 2 h, followed by 7:3 acetone/epon overnight at RT, 100% epon for 2 h and finally 100% epon for 48 h at 60 °C for polymerization. The sections were supported on copper grids. The 70 nm sections were post-stained in Uranyl acetate and lead citrate, and the stained sections were imaged using a transmission electron microscope (JEM-1230) operated at 160 kV.

### Propidium iodide staining and FACS analysis

To quantify hES cells viability, single-cell suspensions were prepared using trypsin, washed in PBS and then fixed in 70% ethanol. Cells were incubated in 0.1 mg/ml PI (Sigma) for 30 min at room temperature, washed and analyzed by flow cytometry.

### Hexokinase activity assay

For HK1 activity measurement *in vitro*, HEK293 *Smad5* KO cells transiently expressing *flag-HK1* were lysed in IP buffer and the lysates centrifuged at highest speed. Supernatants were incubated with anti-Flag M2-conjugated agarose beads for 6 h at 4 °C. The M2 beads were then washed three times with wash buffer (50 mM Tris-HCl pH 7.4, 150 mM NaCl, 1 mM EDTA, 1% Triton X-100, 5% glycerol). The pulled-down flag-HK1 protein was then eluted from the beads with elution buffer (50 mM Tris-HCl pH 7.4, 150 mM NaCl, 150 ng/μl 3×Flag peptide, 1 mM PMSF) for 1 h at 4 °C. For purification of GFP-Smad5, GFP-Smad1, GFP-Smad8 and GFP, HEK293 *Smad5* KO cells were transfected with *GFP*, *GFP-Smad5*, *GFP-Smad1* or *GFP-Smad8* plasmids. Cells were lysed in IP buffer, after centrifugation, supernatants were mixed with 1 μg primary GFP antibody (A6455, life technologies) and 30 μl of 50% slurry Protein-G Sepharose beads (Roche) for 6 h at 4 °C. The beads were then washed three times with high salt buffer (50 mM Tris-HCl pH 7.4, 300 mM NaCl, 1 mM EDTA, 1% Triton X-100, 5% glycerol). The purified GFP, GFP-Smad5, GFP-Smad1 and GFP-Smad8 proteins were then incubated with eluted flag-HK1 protein in HK Assay Buffer for 1 h at 4 °C. HK1 activity was measured with Hexokinase Colorimetric Assay Kit (Biovision).

The HK activity in HEK293, Hela and hES were measured with Hexokinase Colorimetric Assay (Biovision), according to the manufacturer's instructions.

### Statistical analysis

Data are presented as mean ± SEM. Unpaired two-tailed Student's *t*-test for two groups. One-way ANOVA was used for multiple comparisons. Statistical significance is considered at *P*-values below 0.05. ^*^P < 0.05; ^**^P < 0.005.

### Data availability

All high-throughput data have been deposited at the Gene Expression Omnibus under accession number GSE99687.

## Author Contributions

ZX, LL, XB, YY, ZQ and JG conceived the study. FY performed most of the experiments. LZ, CZ, XX, WY, ZY, FB, LY, SL, HY, ZX, ZB, FH, ML and ZQ helped to setup the gene targeting system, culture hES and part of the molecular study. XM performed the MS/MS analysis and HK activity assay. ZX, DY and JC performed the bioinformatics analysis. FY, XB, LL, DT-K and XZ wrote the manuscript.

## Competing Financial Interests

The authors declare no competing financial interests.

## Supplementary Information

Supplementary information, Figure S1 Smad5 accumulates in cytoplasm at low temperature.

Supplementary information, Figure S2 Oxidative stress, membrane potential and signaling modifiers could not obviously affect Smad5, Smad1 or Smad8 subcellular distribution under normal or low temperature culture conditions.

Supplementary information, Figure S3 Smad5 responses to pHe and osmolarity fluctuations.

Supplementary information, Figure S4 Smad5 subjects to accelerated CRM1-dependent nuclear export at low temperature and three NESs are involved in its rapid nuclear export.

Supplementary information, Figure S5 Environmental cues triggered Smad5 translocation is BMP signaling-independent.

Supplementary information, Figure S6 Temperature, pHe and osmolarity fluctuations can induce pHi changes and Smad5 MH1 domain is responsible for pHi sensing.

Supplementary information, Figure S7 Transcriptome analysis of WT and Smad5 KO hES.

Supplementary information, Figure S8 Transcriptome analysis of WT and LDN treated hES.

Supplementary information, Figure S9 Chronic metabolic adaptation of Smad5 KO hES.

Supplementary information, Figure S10 Smad5 shuttling could not regulate metabolism-related gene expression.

Supplementary information, Figure S11 Smad5 KO reduces glycolysis.

Supplementary information, Figure S12 Smad5 KO induces irreversible mitochondrial morphology changes.

Supplementary information, Figure S13 Smad5 KO impaired mitochondrial respiration.

Supplementary information, Figure S14 Increased pHi during neural differentiation.

Supplementary information, Figure S15 Smad5 interacts with HK1.

Supplementary information, Movie S1 Timelapse video recording of nuclear export of GFP-Smad5 at 25°C. *GFP-Smad5* HEK293 cells were cultured at 37°C, then the cells were placed at 25°C to record the video.

Supplementary information, Movie S2 Timelapse video recording of nuclear export of GFP-Smad5 at basic extracellular pH. *GFP-Smad5* HEK293 cells were cultured at 37°C, then the cells were placed at basic extracellular pH conditions to record the video.

Supplementary information, Movie S3 Timelapse video recording of nuclear export of GFP-Smad5 at hypertonic conditions. *GFP-Smad5* HEK293 cells were cultured at 37°C, then the cells were placed at hypertonic conditions to record the video.

Supplementary information, Movie S4 Timelapse video recording of cytosol to nuclear translocation of GFP-Smad5 at acidic extracellular pH. *GFP-Smad5* HEK293 cells were cultured at 37°C, then the cells were placed at acidic extracellular pH conditions to record the video.

Supplementary information, Movie S5 Timelapse video recording of cytosol to nuclear translocation of GFP-Smad5 at hypotonic conditions. *GFP-Smad5* HEK293 cells were cultured at 37°C, then the cells were placed at hypotonic conditions to record the video.

Supplementary information, Table S1 Smad5 (cytosolic) pull down proteins

## References

[bib1] Salvi A, Quillan JM, Sadee W. Monitoring intracellular pH changes in response to osmotic stress and membrane transport activity using 5-chloromethylfluorescein. AAPS PharmSci 2002; 4:E21. 12645993 10.1208/ps040421 PMC2751310

[bib2] Casey JR, Grinstein S, Orlowski J. Sensors and regulators of intracellular pH. Nat Rev Mol Cell Biol 2010; 11:50–61. 19997129 10.1038/nrm2820

[bib3] Korolchuk VI, Saiki S, Lichtenberg M, et al. Lysosomal positioning coordinates cellular nutrient responses. Nat Cell Biol 2011; 13:453–460. 21394080 10.1038/ncb2204 PMC3071334

[bib4] Clement MV, Hirpara JL, Pervaiz S. Decrease in intracellular superoxide sensitizes Bcl-2-overexpressing tumor cells to receptor and drug-induced apoptosis independent of the mitochondria. Cell Death Differ 2003; 10:1273–1285. 12894215 10.1038/sj.cdd.4401302

[bib5] Schmierer B, Hill CS. TGFbeta-SMAD signal transduction: molecular specificity and functional flexibility. Nat Rev Mol Cell Biol 2007; 8:970–982. 18000526 10.1038/nrm2297

[bib6] Derynck R, Zhang Y, Feng XH. Smads: transcriptional activators of TGF-beta responses. Cell 1998; 95:737–740. 9865691 10.1016/s0092-8674(00)81696-7

[bib7] Massague J, Seoane J, Wotton D. Smad transcription factors. Genes Dev 2005; 19:2783–2810. 16322555 10.1101/gad.1350705

[bib8] Shi Y, Massague J. Mechanisms of TGF-beta signaling from cell membrane to the nucleus. Cell 2003; 113:685–700. 12809600 10.1016/s0092-8674(03)00432-x

[bib9] Moustakas A, Souchelnytskyi S, Heldin CH. Smad regulation in TGF-beta signal transduction. J Cell Sci 2001; 114:4359–4369. 11792802 10.1242/jcs.114.24.4359

[bib10] Chang H, Lau AL, Matzuk MM. Studying TGF-beta superfamily signaling by knockouts and knockins. Mol Cell Endocrinol 2001; 180:39–46. 11451570 10.1016/s0303-7207(01)00513-5

[bib11] Kaivo-oja N, Jeffery LA, Ritvos O, Mottershead DG. Smad signalling in the ovary. Reprod Biol Endocrinol 2006; 4:21.16611366

[bib12] Kumar S, Pan CC, Shah N, et al. Activation of Mitofusin2 by Smad2-RIN1 complex during mitochondrial fusion. Mol Cell 2016; 62:520–531. 27184078 10.1016/j.molcel.2016.04.010 PMC4877164

[bib13] Sun Y, Zhou J, Liao X, et al. Disruption of Smad5 gene induces mitochondria-dependent apoptosis in cardiomyocytes. Exp Cell Res 2005; 306:85–93. 15878335 10.1016/j.yexcr.2005.02.012

[bib14] Stade K, Ford CS, Guthrie C, Weis K. Exportin 1 (Crm1p) is an essential nuclear export factor. Cell 1997; 90:1041–1050. 9323132 10.1016/s0092-8674(00)80370-0

[bib15] Adachi Y, Yanagida M. Higher order chromosome structure is affected by cold-sensitive mutations in a *Schizosaccharomyces pombe* gene crm1^+^ which encodes a 115-kD protein preferentially localized in the nucleus and its periphery. J Cell Biol 1989; 108:1195–1207. 2647765 10.1083/jcb.108.4.1195 PMC2115495

[bib16] Fukuda M, Asano S, Nakamura T, et al. CRM1 is responsible for intracellular transport mediated by the nuclear export signal. Nature 1997; 390:308–311. 9384386 10.1038/36894

[bib17] Kutay U, Guttinger S. Leucine-rich nuclear-export signals: born to be weak. Trends Cell Biol 2005; 15:121–124. 15752974 10.1016/j.tcb.2005.01.005

[bib18] Attisano L, Wrana JL. Signal transduction by the TGF-beta superfamily. Science 2002; 296:1646–1647. 12040180 10.1126/science.1071809

[bib19] Miyauchi K, Marin M, Melikyan GB. Visualization of retrovirus uptake and delivery into acidic endosomes. Biochem J 2011; 434:559–569. 21175427 10.1042/BJ20101588 PMC3249399

[bib20] Miesenbock G, De Angelis DA, Rothman JE. Visualizing secretion and synaptic transmission with pH-sensitive green fluorescent proteins. Nature 1998; 394:192–195. 9671304 10.1038/28190

[bib21] Morimoto YV, Kojima S, Namba K, Minamino T. M153R mutation in a pH-sensitive green fluorescent protein stabilizes its fusion proteins. PLoS One 2011; 6:e19598. 21559297 10.1371/journal.pone.0019598 PMC3086926

[bib22] Chi L, Fan B, Feng D, et al. The dorsoventral patterning of human forebrain follows an activation/transformation model. Cereb Cortex 2017; 27:2941–2954. 27226442 10.1093/cercor/bhw152

[bib23] Liu L, Liu X, Ren X, et al. Smad2 and Smad3 have differential sensitivity in relaying TGFbeta signaling and inversely regulate early lineage specification. Sci Rep 2016; 6:21602. 26905010 10.1038/srep21602 PMC4764856

[bib24] Wallace DC. Mitochondria and cancer. Nat Rev Cancer 2012; 12:685–698. 23001348 10.1038/nrc3365 PMC4371788

[bib25] Nunnari J, Suomalainen A. Mitochondria: in sickness and in health. Cell 2012; 148:1145–1159. 22424226 10.1016/j.cell.2012.02.035 PMC5381524

[bib26] Chambers SM, Fasano CA, Papapetrou EP, Tomishima M, Sadelain M, Studer L. Highly efficient neural conversion of human ES and iPS cells by dual inhibition of SMAD signaling. Nat Biotechnol 2009; 27:275–280. 19252484 10.1038/nbt.1529 PMC2756723

[bib27] Ulmschneider B, Grillo-Hill BK, Benitez M, Azimova DR, Barber DL, Nystul TG. Increased intracellular pH is necessary for adult epithelial and embryonic stem cell differentiation. J Cell Biol 2016; 215:345–355. 27821494 10.1083/jcb.201606042 PMC5100294

[bib28] Chi L, Fan B, Zhang K, et al. Targeted differentiation of regional ventral neuroprogenitors and related neuronal subtypes from human pluripotent stem cells. Stem Cell Rep 2016; 7:941–954. 10.1016/j.stemcr.2016.09.003 PMC5106484 27720902

[bib29] Bustamante E, Morris HP, Pedersen PL. Energy metabolism of tumor cells. Requirement for a form of hexokinase with a propensity for mitochondrial binding. J Biol Chem 1981; 256:8699–8704. 7263678

[bib30] Cairns RA, Harris IS, Mak TW. Regulation of cancer cell metabolism. Nat Rev Cancer 2011; 11:85–95. 21258394 10.1038/nrc2981

[bib31] Moore B, Zhou L, Rolland F, et al. Role of the Arabidopsis glucose sensor HXK1 in nutrient, light, and hormonal signaling. Science 2003; 300:332–336. 12690200 10.1126/science.1080585

[bib32] Greiner EF, Guppy M, Brand K. Glucose is essential for proliferation and the glycolytic enzyme induction that provokes a transition to glycolytic energy production. J Biol Chem 1994; 269:31484–31490. 7989314

[bib33] Harland R. Neural induction. Curr Opin Genet Dev 2000; 10:357–362. 10889069 10.1016/s0959-437x(00)00096-4

[bib34] Chen D, Zhao M, Mundy GR. Bone morphogenetic proteins. Growth Factors 2004; 22:233–241. 15621726 10.1080/08977190412331279890

[bib35] Parks SK, Chiche J, Pouyssegur J. pH control mechanisms of tumor survival and growth. J Cell Physiol 2011; 226:299–308. 20857482 10.1002/jcp.22400

[bib36] Faust M, Montenarh M. Subcellular localization of protein kinase CK2. A key to its function? *Cell Tissue Res* 2000; 301:329–340. 10994779 10.1007/s004410000256

[bib37] Reguly T, Wrana JL. In or out? The dynamics of Smad nucleocytoplasmic shuttling. Trends Cell Biol 2003; 13:216–220. 12742164 10.1016/s0962-8924(03)00075-8

[bib38] Brunet A, Bonni A, Zigmond MJ, et al. Akt promotes cell survival by phosphorylating and inhibiting a Forkhead transcription factor. Cell 1999; 96:857–868. 10102273 10.1016/s0092-8674(00)80595-4

[bib39] Agarraberes FA, Dice JF. Protein translocation across membranes. Biochim Biophys Acta 2001; 1513:1–24. 11427190 10.1016/s0304-4157(01)00005-3

[bib40] Rapoport TA. Protein translocation across the eukaryotic endoplasmic reticulum and bacterial plasma membranes. Nature 2007; 450:663–669. 18046402 10.1038/nature06384

[bib41] Schnell DJ, Hebert DN. Protein translocons: multifunctional mediators of protein translocation across membranes. Cell 2003; 112:491–505. 12600313 10.1016/s0092-8674(03)00110-7

[bib42] Pouyssegur J, Franchi A, L'Allemain G, Paris S. Cytoplasmic pH, a key determinant of growth factor-induced DNA synthesis in quiescent fibroblasts. FEBS Lett 1985; 190:115–119. 4043390 10.1016/0014-5793(85)80439-7

[bib43] Brooks C, Ketsawatsomkron P, Sui Y, et al. Acidic pH inhibits ATP depletion-induced tubular cell apoptosis by blocking caspase-9 activation in apoptosome. Am J Physiol Renal Physiol 2005; 289:F410–419. 15755925 10.1152/ajprenal.00440.2004

[bib44] Loiselle FB, Casey JR. Measurement of intracellular pH. Methods Mol Biol 2010; 637:311–331. 20419443 10.1007/978-1-60761-700-6_17

[bib45] Zhang X, Huang CT, Chen J, et al. Pax6 is a human neuroectoderm cell fate determinant. Cell Stem Cell 2010; 7:90–100. 20621053 10.1016/j.stem.2010.04.017 PMC2904346

[bib46] Chen M, Huang SL, Zhang XQ, et al. Reversal effects of pantoprazole on multidrug resistance in human gastric adenocarcinoma cells by down-regulating the V-ATPases/mTOR/HIF-1alpha/P-gp and MRP1 signaling pathway *in vitro* and *in vivo*. J Cell Biochem 2012; 113:2474–2487. 22396185 10.1002/jcb.24122 PMC3762681

[bib47] Sanjana NE, Cong L, Zhou Y, Cunniff MM, Feng G, Zhang F. A transcription activator-like effector toolbox for genome engineering. Nat Protoc 2012; 7:171–192. 22222791 10.1038/nprot.2011.431 PMC3684555

[bib48] Hockemeyer D, Wang H, Kiani S, et al. Genetic engineering of human pluripotent cells using TALE nucleases. Nat Biotechnol 2011; 29:731–734. 21738127 10.1038/nbt.1927 PMC3152587

[bib49] Locasale JW, Grassian AR, Melman T, et al. Phosphoglycerate dehydrogenase diverts glycolytic flux and contributes to oncogenesis. Nat Genet 2011; 43:869–874. 21804546 10.1038/ng.890 PMC3677549

