# Supplementary material for: Smad5 acts as an intracellular pH messenger and maintains bioenergetic homeostasis
Source: Cell Res. 2017 Jul 4;27(9):1083–99. doi: 10.1038/cr.2017.85 (PMC5587853; doi:10.1038/cr.2017.85)
Supplement: Supplementary information, Figure S1 — Smad5 accumulates in cytoplasm at low temperature. [file cr201785x1.pdf]

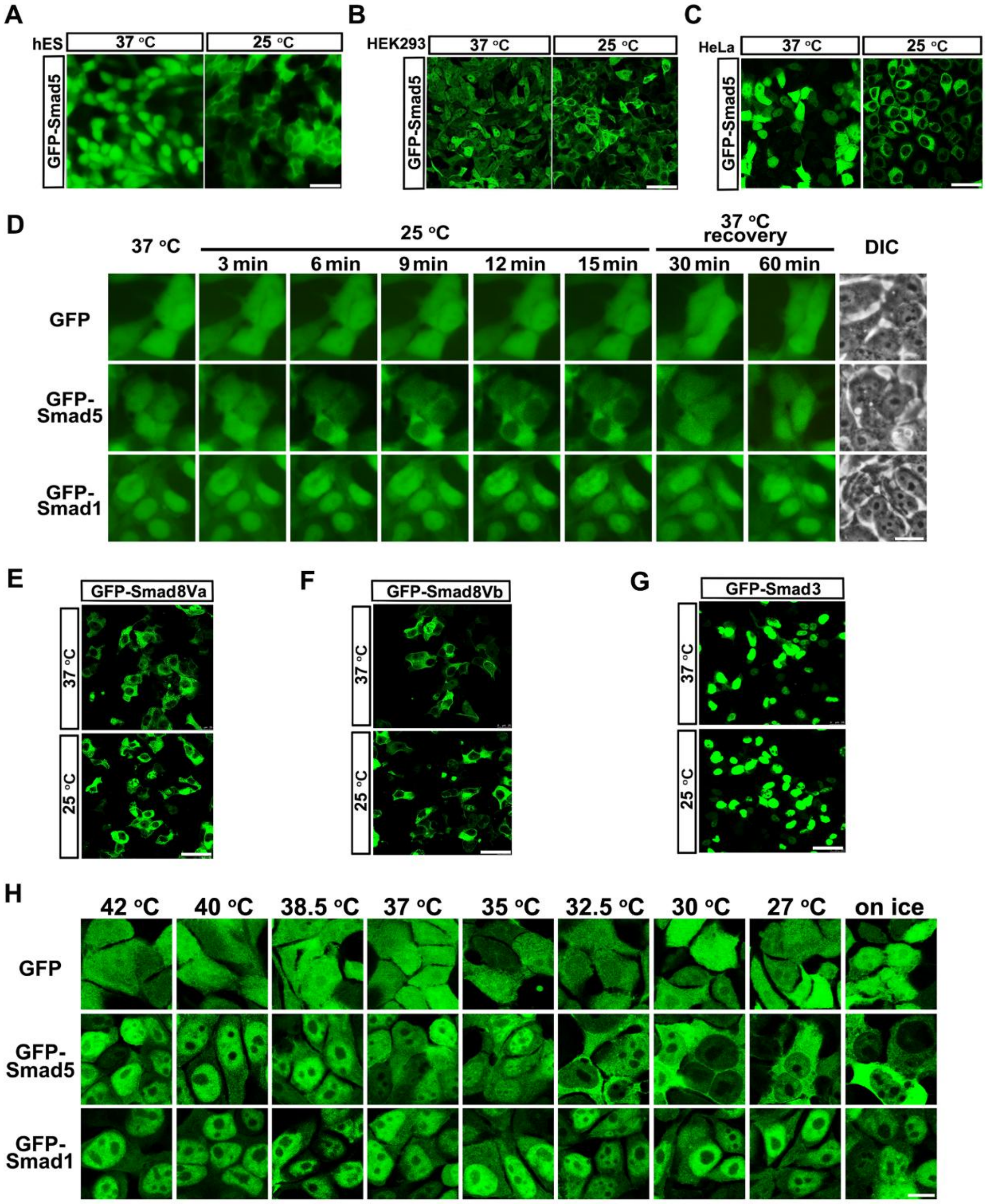

A
hES
37 °C
25 °C
GFP-Smad5
B
HEK293
37 °C
25 °C
GFP-Smad5
C
HeLa
37 °C
25 °C
GFP-Smad5
D
37 °C
25 °C
3 min
6 min
9 min
12 min
15 min
37 °C recovery
30 min
60 min
DIC
GFP
GFP-Smad5
GFP-Smad1
E
GFP-Smad8Va
37 °C
25 °C
F
GFP-Smad8Vb
37 °C
25 °C
G
GFP-Smad3
37 °C
25 °C
H
42 °C
40 °C
38.5 °C
37 °C
35 °C
32.5 °C
30 °C
27 °C
on ice
GFP
GFP-Smad5
GFP-Smad1

Supplementary information, Figure S1. Smad5 accumulates in cytoplasm at low temperature. **(A-C)** In hES **(A)**, HEK293 **(B)** and HeLa **(C)** cells, GFP-Smad5 shows apparent cytoplasmic accumulation at 25°C. Scale bars, 50 µm. **(D)** Timelapse experiments show that placing the cells at 25°C gradually drives GFP-Smad5 into the cytoplasm, but neither GFP nor GFP-Smad1 is affected. Bright field images show nuclear morphology. Scale bar, 10 µm. **(E-G)** The nucleocytoplasmic distribution of other downstream signaling mediators of TGFβ superfamily, Smad8a **(E)**, Smad8b **(F)** and Smad3 **(G)**, remains unchanged upon temperature shift. Scale bars, 50 µm. **(H)** Confocal images show that GFP-Smad5 is largely distributed in the cytoplasm at a temperature lower than 32.5^{o}C. Scale bar, 10 µm.
