# Supplementary material for: Smad5 acts as an intracellular pH messenger and maintains bioenergetic homeostasis
Source: Cell Res. 2017 Jul 4;27(9):1083–99. doi: 10.1038/cr.2017.85 (PMC5587853; doi:10.1038/cr.2017.85)
Supplement: Supplementary information, Figure S2 — Oxidative stress, membrane potential and signaling modifiers could not obviously affect Smad5, Smad1 or Smad8 subcellular distribution under normal or low temperature culture conditions. [file cr201785x2.pdf]

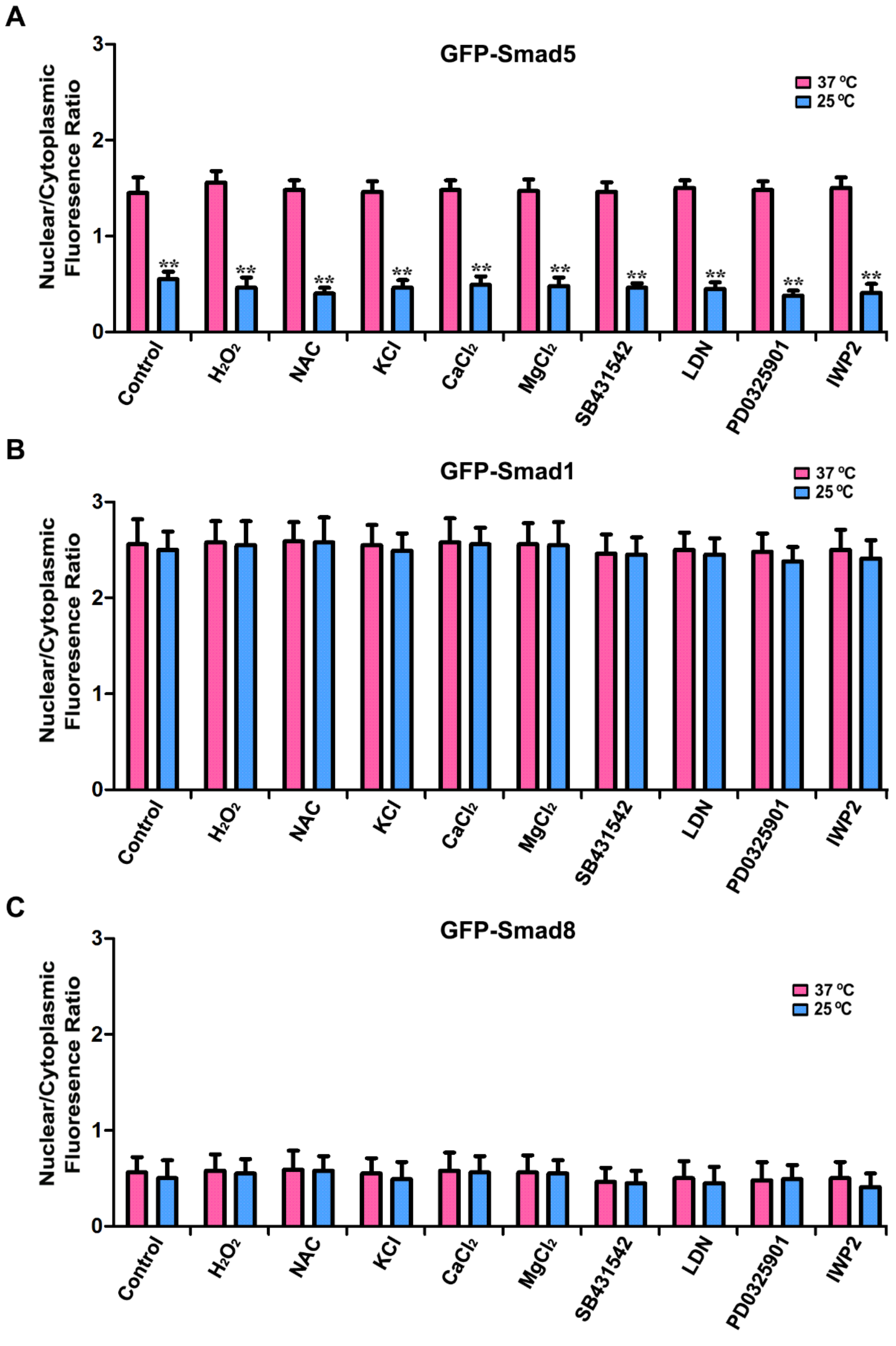
A
GFP-Smad5
Nuclear/Cytoplasmic Fluoresence Ratio
37 ºC
25 ºC
**
Control
H2O2
NAC
KCl
CaCl2
MgCl2
SB431542
LDN
PD0325901
IWP2
B
GFP-Smad1
C
GFP-Smad8

Supplementary information, Figure S2. Oxidative stress, membrane potential and signaling modifiers could not obviously affect Smad5, Smad1 or Smad8 subcellular distribution under normal or low temperature culture conditions. NAC, N-acetylcysteine (anti-oxidant); SB431542, a selective inhibitor of TGFβ superfamily type I activin receptor-like kinase (ALK) receptors; LDN193189, a selective inhibitor of BMP type I receptors; PD0325901, a selective inhibitor of MEK and subsequently inhibits ERK signaling; IWP2, a selective inhibitor of canonical Wnt signaling. Data are presented as mean ± s.e.m of three independent experiments. **p < 0.01.
