# Supplementary material for: Smad5 acts as an intracellular pH messenger and maintains bioenergetic homeostasis
Source: Cell Res. 2017 Jul 4;27(9):1083–99. doi: 10.1038/cr.2017.85 (PMC5587853; doi:10.1038/cr.2017.85)
Supplement: Supplementary information, Figure S3 — Smad5 responses to pHe and osmolarity fluctuations. [file cr201785x3.pdf]

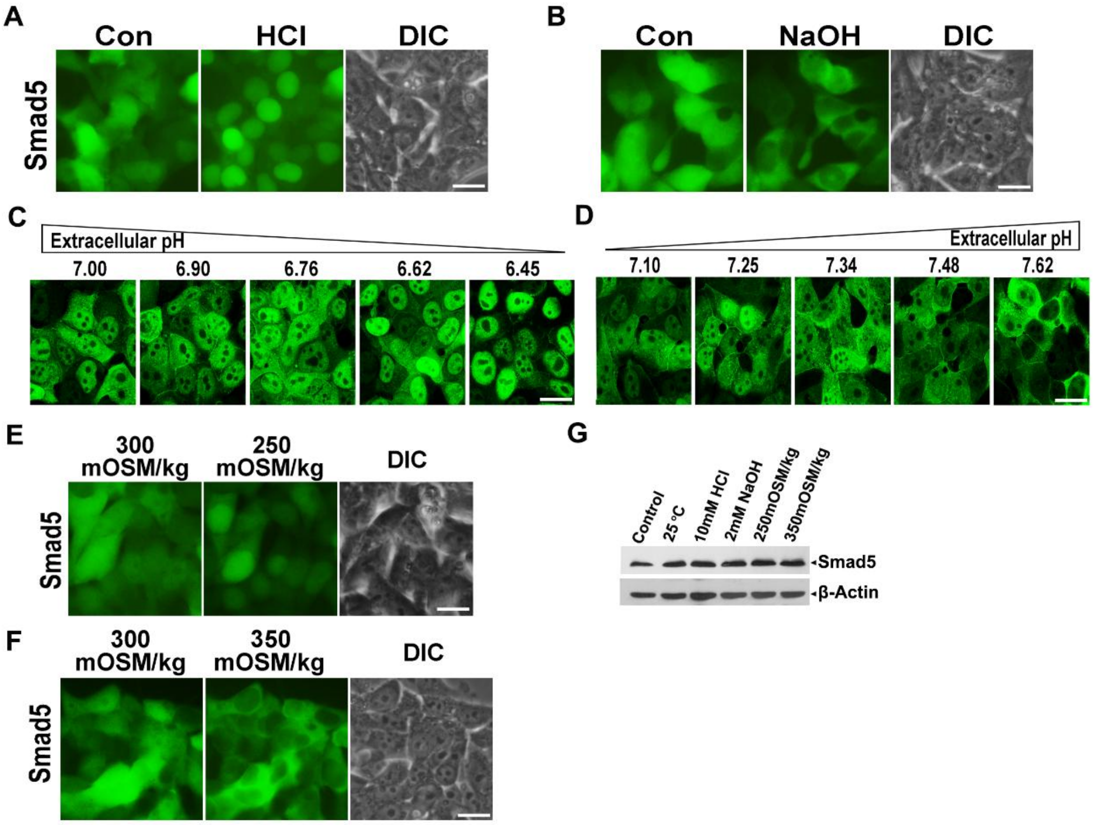

A
Con
HCl
DIC
Smad5
B
Con
NaOH
DIC
C
Extracellular pH
7.00
6.90
6.76
6.62
6.45
D
Extracellular pH
7.10
7.25
7.34
7.48
7.62
E
300 mOSM/kg
250 mOSM/kg
DIC
Smad5
F
300 mOSM/kg
350 mOSM/kg
DIC
Smad5
G
Control
25°C
10mM HCl
2mM NaOH
250mOSM/kg
350mOSM/kg
Smad5
β-Actin

Supplementary information, Figure S3. Smad5 responses to pHe and osmolarity fluctuations. **(A)** Treatment of *GFP-Smad5* HEK293 cells with 10 mM hydrochloric acid for 10 min at 37°C leads to the rapid nuclear accumulation of Smad5. Scale bar, 10 μm. **(B)** Treatment of the cells with 2 mM sodium hydroxide for 2 min at 37°C induces rapid translocation of Smad5 into the cytoplasm. Scale bar, 10 μm. **(C, D)** Confocal images show more GFP-Smad5 nuclear accumulation at lower pHe, while cytoplasmic accumulation at higher pHe. Scale bars, 10 μm. **(E)** Smad5 shows more obvious nuclear accumulation when cells are exposed to hypotonic solutions at an osmolarity of 250 mOSM/kg. Scale bar, 10 μm. **(F)** Smad5 translocates into the cytoplasm when extracellular osmolarity is increased to 350 mOSM/kg. Scale bar, 10 μm. **(G)** Western blotting shows the total level of Smad5 under various temperature, pHe and osmolarity conditions.
