# Supplementary material for: Smad5 acts as an intracellular pH messenger and maintains bioenergetic homeostasis
Source: Cell Res. 2017 Jul 4;27(9):1083–99. doi: 10.1038/cr.2017.85 (PMC5587853; doi:10.1038/cr.2017.85)
Supplement: Supplementary information, Figure S4 — Smad5 subjects to accelerated CRM1-dependent nuclear export at low temperature and three NESs are involved in its rapid nuclear export. [file cr201785x4.pdf]

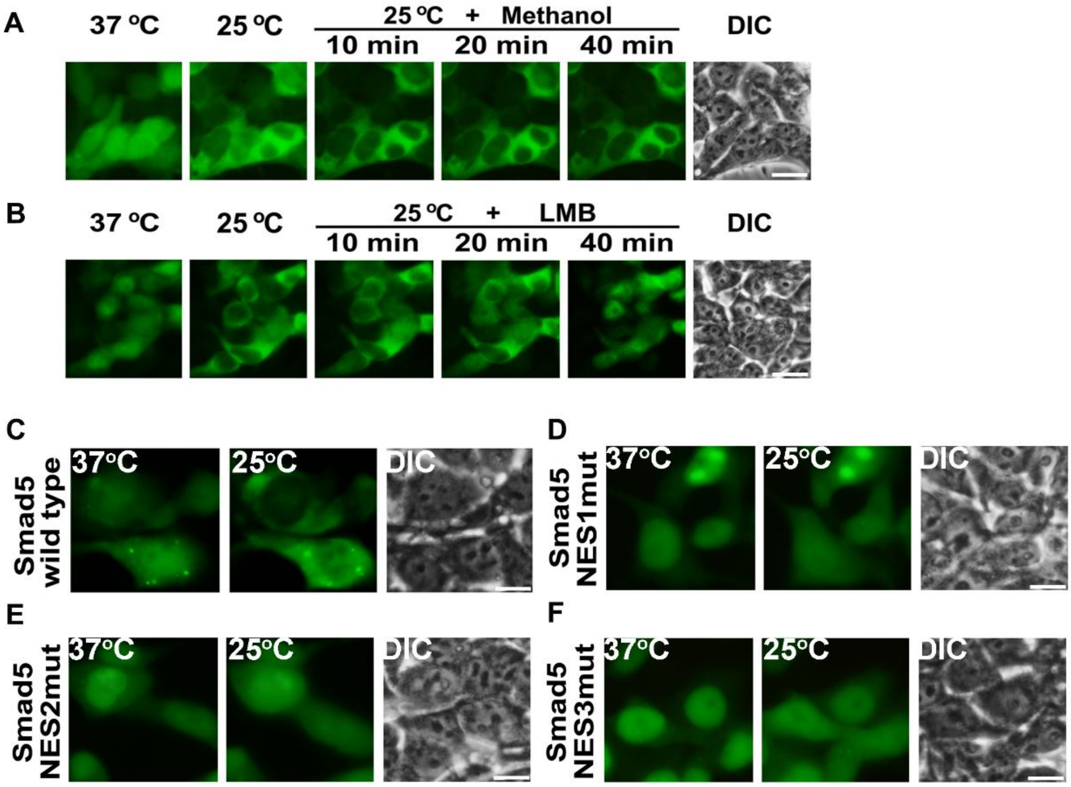
A
37 ºC
25 ºC
25 ºC + Methanol
10 min
20 min
40 min
DIC
B
37 ºC
25 ºC
25 ºC + LMB
10 min
20 min
40 min
DIC
C
Smad5 wild type
37°C
25°C
DIC
D
Smad5 NES1mut
37°C
25°C
DIC
E
Smad5 NES2mut
37°C
25°C
DIC
F
Smad5 NES3mut
37°C
25°C
DIC

Supplementary information, Figure S4. Smad5 subjects to accelerated CRM1-dependent nuclear export at low temperature and three NESs are involved in its rapid nuclear export. **(A, B)** *GFP-Smad5* HEK293 cell lines were placed at 25°C for 30 min in order to transport Smad5 from nucleus to cytoplasm completely. The cells were then treated with methanol or 5ng/ml LMB for 40 min at 25°C. Fluorescence study now shows exclusive nuclear distribution of Smad5 under LMB treatment. Scale bars, 10 μm. **(C)** HEK293 cells with transient *GFP-Smad5* overexpression show nuclear export of Smad5 at 25°C. Scale bar, 10 μm. **(D-F)** Individual mutation of the three NESs promotes nuclear accumulation of Smad5 at 37°C and all three mutants exhibit a slower nuclear export rate upon low temperature challenge. Scale bars, 10 μm.
