# Supplementary material for: Smad5 acts as an intracellular pH messenger and maintains bioenergetic homeostasis
Source: Cell Res. 2017 Jul 4;27(9):1083–99. doi: 10.1038/cr.2017.85 (PMC5587853; doi:10.1038/cr.2017.85)
Supplement: Supplementary information, Figure S5 — Environmental cues triggered Smad5 translocation is BMP signaling-independent. [file cr201785x5.pdf]

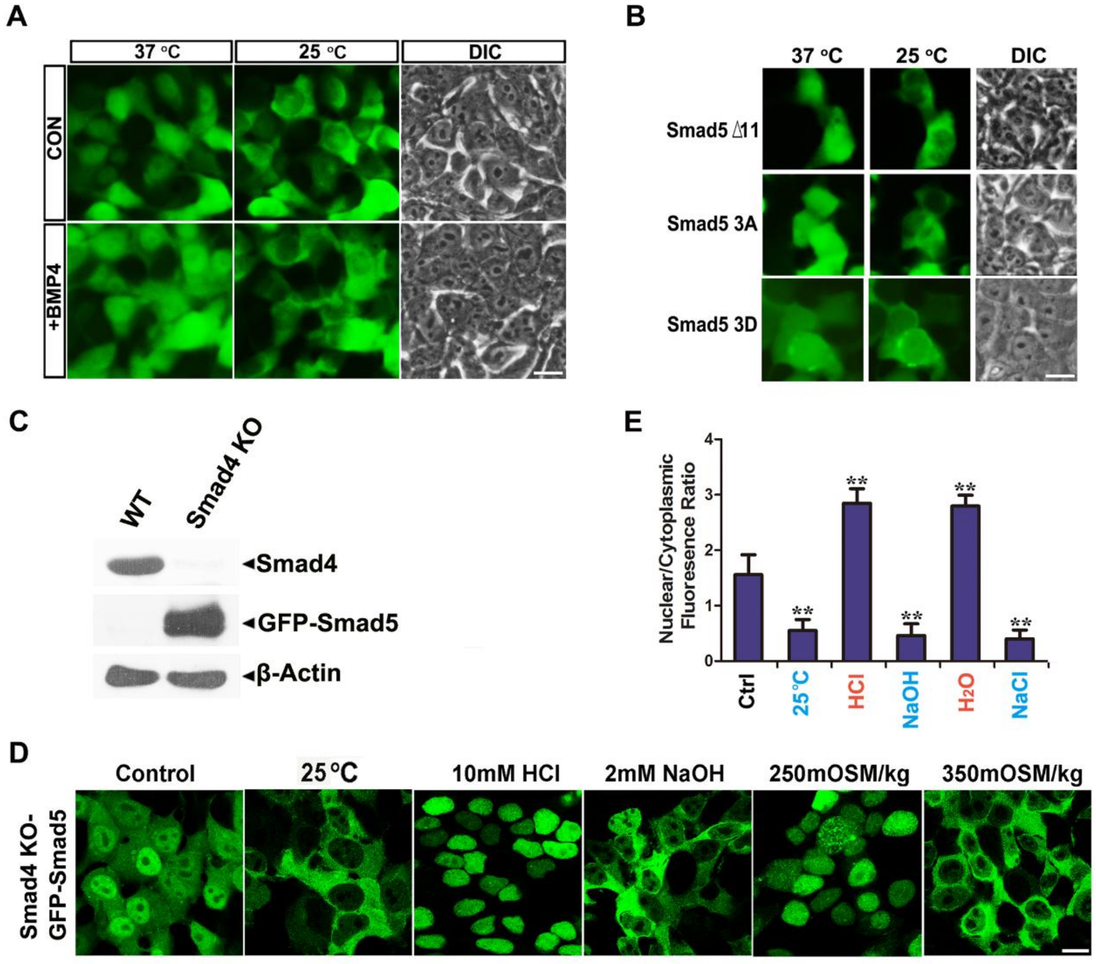

A
37 °C
25 °C
DIC
CON
+BMP4
B
37 °C
25 °C
DIC
Smad5 △11
Smad5 3A
Smad5 3D
C
WT
Smad4 KO
Smad4
GFP-Smad5
β-Actin
E
Nuclear/Cytoplasmic Fluoresence Ratio
0
1
2
3
4
**
Ctrl
25°C
HCl
NaOH
H2O
NaCl
D
Control
25°C
10mM HCl
2mM NaOH
250mOSM/kg
350mOSM/kg
Smad4 KO-GFP-Smad5

Supplementary information, Figure S5. Environmental cues-triggered Smad5 translocation is BMP signaling-independent. **(A)** Low temperature could still induce GFP-Smad5 cytoplasmic accumulation when cells were pretreated with BMP4 at 37°C for 1 hr. **(B)** Deletion of the C-terminal 11 amino acids containing SSVS motif (Smad5 Δ11), or mutation of the three potential phosphorylation sites to alanine (3A) or aspartic acid (3D) does not affect low temperature-mediated Smad5 nucleocytoplasmic shuttling. Scale bar, 10 µm. **(C)** Western blotting shows that *GFP-Smad5* is overexpressed in *Smad4* KO HEK293 cells. **(D)** *Smad4* KO does not affect Smad5 nucleocytoplasmic shuttling triggered by fluctuations of temperature, pHe and osmolarity. Scale bar, 10 µm. **(E)** Average fluorescence quantification of nuclear and cytoplasmic portions of GFP-Smad5 at various conditions in **D** (n=30; data are presented as mean ± s.e.m. **p < 0.01).
