# Supplementary material for: Smad5 acts as an intracellular pH messenger and maintains bioenergetic homeostasis
Source: Cell Res. 2017 Jul 4;27(9):1083–99. doi: 10.1038/cr.2017.85 (PMC5587853; doi:10.1038/cr.2017.85)
Supplement: Supplementary information, Figure S6 — Temperature, pHe and osmolarity fluctuations can induce pHi changes and Smad5 MH1 domain is responsible for pHi sensing. [file cr201785x6.pdf]

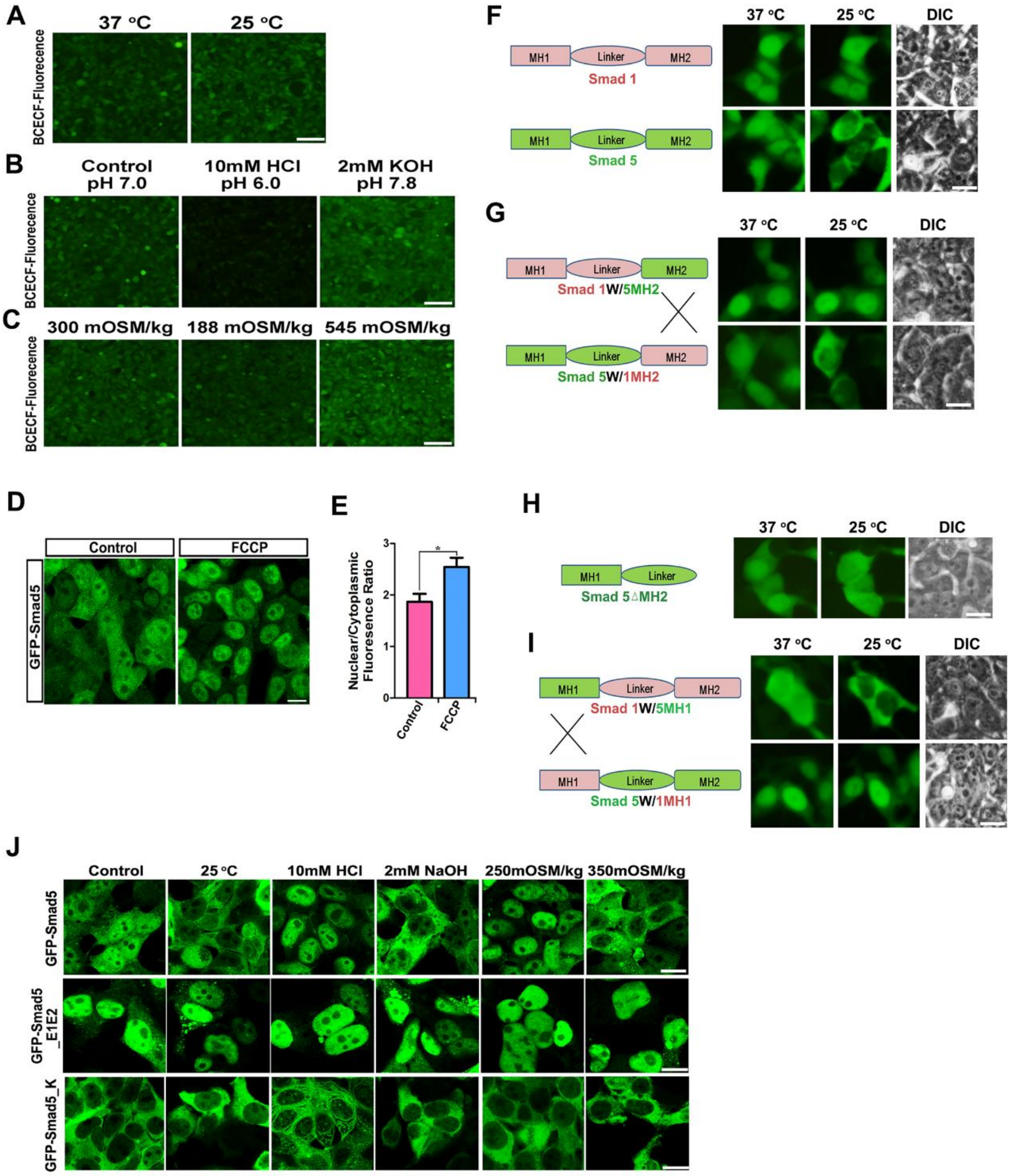
A
37 °C
25 °C
BCECF-Fluorecence
B
Control pH 7.0
10mM HCl pH 6.0
2mM KOH pH 7.8
C
300 mOSM/kg
188 mOSM/kg
545 mOSM/kg
D
Control
FCCP
GFP-Smad5
E
Nuclear/Cytoplasmic Fluoresence Ratio
Control
FCCP
F
MH1
Linker
MH2
Smad 1
Smad 5
DIC
G
Smad 1W/5MH2
Smad 5W/1MH2
H
Smad 5△MH2
I
Smad 1W/5MH1
Smad 5W/1MH1
J
Control
25 °C
10mM HCl
2mM NaOH
250mOSM/kg
350mOSM/kg
GFP-Smad5
GFP-Smad5_E1E2
GFP-Smad5_K

Supplementary information, Figure S6. Temperature, pHe and osmolarity fluctuations can induce pHi changes and Smad5 MH1 domain is responsible for pHi sensing. **(A)** Comparison of the fluorescence intensity of BCECF in HEK293 cells at 37°C and 25°C. Scale bar, 50 μm. **(B)** Comparison of the fluorescence intensity of BCECF at various extracellular pH solutions. Scale bar, 50 μm. **(C)** Representative images of intracellular BCECF fluorescence at various extracellular osmolarity. Scale bar, 50 μm. **(D)** FCCP treatment of *GFP-Smad5* HEK293 cells for 30 min promoted Smad5 nuclear accumulation. Scale bar, 10 μm. **(E)** Average fluorescence quantification of nuclear and cytoplasmic localized GFP-Smad5 in **D**. (n=30; data are mean ± s.e.m. *$p < 0.05$). **(F)** Smad5, but not Smad1 transiently expressed in HEK293 cells shows quick nuclear export at 25°C. Scale bar, 10 μm. **(G)** Interchanging MH2 domains between Smad1 and Smad5 does not affect their own subcellular distribution at either 37 ^{o}C or 25 ^{o}C. Scale bar, 10 μm. **(H)** Mutant Smad5 with MH2 domain deleted shows moderate nuclear export 25 min after placing the cells at 25°C. Scale bar, 10 μm. **(I)** GFP-Smad5 with Smad1 MH1 domain resides in nucleus at both 37°C and 25°C. Whereas, GFP-Smad1 harboring Smad5 MH1 domain shows low temperature-induced nuclear export. Scale bar, 10 μm. **(J)** Nuclear and cytoplasm distribution of GFP-Smad5, GFP-Smad5_E1E2 and GFP-Smad5_K at various temperature, pHe and osmolarity conditions. Scale bars, 10 μm.
