# Supplementary material for: Smad5 acts as an intracellular pH messenger and maintains bioenergetic homeostasis
Source: Cell Res. 2017 Jul 4;27(9):1083–99. doi: 10.1038/cr.2017.85 (PMC5587853; doi:10.1038/cr.2017.85)
Supplement: Supplementary information, Figure S7 — Transcriptome analysis of WT and Smad5 KO hES. [file cr201785x7.pdf]

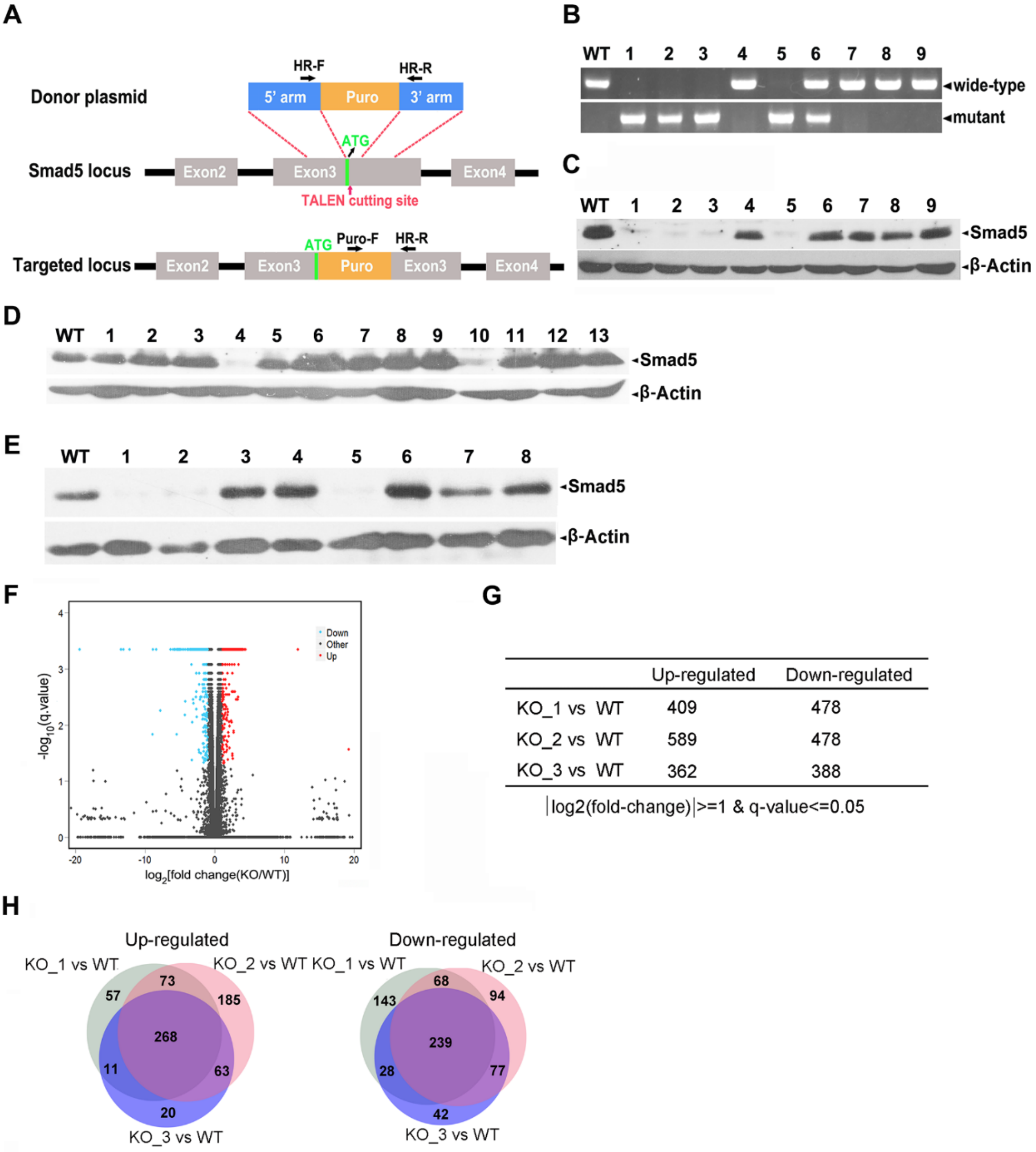
A
Donor plasmid
HR-F
HR-R
5' arm
Puro
3' arm
ATG
Smad5 locus
Exon2
Exon3
Exon4
TALEN cutting site
Targeted locus
Puro-F
B
WT 1 2 3 4 5 6 7 8 9
wide-type
mutant
C
WT 1 2 3 4 5 6 7 8 9
Smad5
β-Actin
D
WT 1 2 3 4 5 6 7 8 9 10 11 12 13
Smad5
β-Actin
E
WT 1 2 3 4 5 6 7 8
Smad5
β-Actin
F
Down
Other
Up
-log10(q.value)
log2[fold change(KO/WT)]
G
Up-regulated Down-regulated
KO_1 vs WT 409 478
KO_2 vs WT 589 478
KO_3 vs WT 362 388
|log2(fold-change)|>=1 & q-value<=0.05
H
Up-regulated
KO_1 vs WT
KO_2 vs WT
KO_3 vs WT
57 73 185 268 11 63 20
Down-regulated
KO_1 vs WT
KO_2 vs WT
KO_3 vs WT
143 68 94 239 28 77 42

Supplementary information, Figure S7. Transcriptome analysis of WT and *Smad5* KO hESCs. **(A)** Schematic depiction of the strategy in construction of *Smad5* KO cell lines. **(B, C)** Genomic PCR **(B)** and western blotting **(C)** show *Smad5* KO efficiency in hESCs. **(D, E)** Western blotting shows *Smad5* KO efficiency in HEK293 **(D)** and HeLa **(E)** cells. **(F)** Volcano plot shows gene expression changes between *Smad5* KO and WT hESCs. Blue dots represent genes that are significantly down-regulated in KO cells, while the red dots represent genes that are significantly up-regulated in KO cells. **(G)** Number of up- or down-regulated genes in triplicate *Smad5* KO hESCs as compared with WT hESCs. **(H)** There is a significant overlap between up- or down-regulated genes in three biological replicates of hESCs.
