# Supplementary material for: Smad5 acts as an intracellular pH messenger and maintains bioenergetic homeostasis
Source: Cell Res. 2017 Jul 4;27(9):1083–99. doi: 10.1038/cr.2017.85 (PMC5587853; doi:10.1038/cr.2017.85)
Supplement: Supplementary information, Figure S8 — Transcriptome analysis of WT and LDN treated hES. [file cr201785x8.pdf]

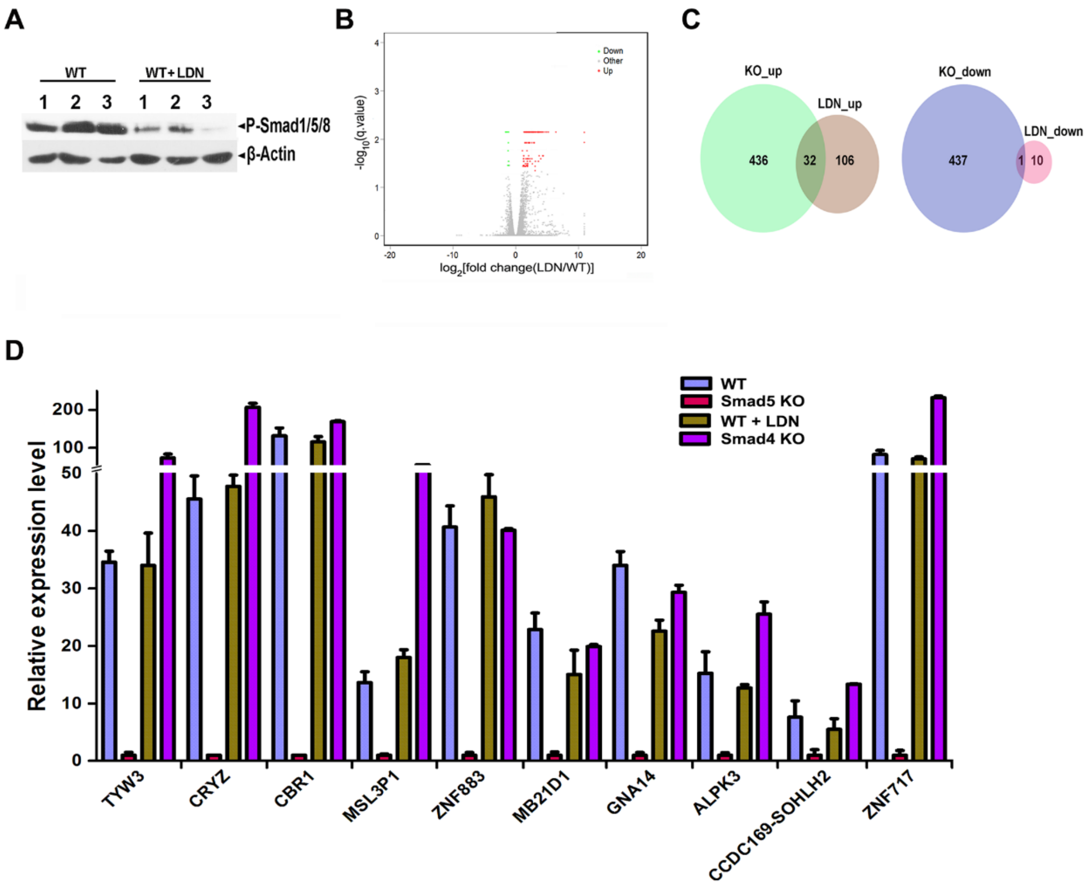
A
WT
WT+LDN
1 2 3 1 2 3
P-Smad1/5/8
β-Actin
B
Down
Other
Up
-log10(p.value)
log2[fold change(LDN/WT)]
C
KO_up
LDN_up
436
32
106
KO_down
LDN_down
437
1
10
D
WT
Smad5 KO
WT + LDN
Smad4 KO
Relative expression level
TYW3
CRYZ
CBR1
MSL3P1
ZNF883
MB21D1
GNA14
ALPK3
CCDC169-SOHLH2
ZNF717

Supplementary information, Figure S8. Transcriptome analysis of WT and LDN treated hESCs. **(A)** Western blotting shows that most BMP-induced Smad1/5/8 SSXS phosphorylation is blocked after 4 days of LDN193189 treatment. **(B)** Transcriptional profiling of LDN193189 treated and untreated hESCs. **(C)** Minimal overlapping between differentially expressed genes in *Smad5* KO and LDN193189 treated hESCs. **(D)** qPCR analysis confirms the gene expression changes in *Smad5* KO hESCs. Each quantification was made from 3 independent images. Data are presented as mean ± s.e.m.
