# Supplementary material for: Smad5 acts as an intracellular pH messenger and maintains bioenergetic homeostasis
Source: Cell Res. 2017 Jul 4;27(9):1083–99. doi: 10.1038/cr.2017.85 (PMC5587853; doi:10.1038/cr.2017.85)
Supplement: Supplementary information, Figure S9 — Chronic metabolic adaptation of Smad5 KO hES. [file cr201785x9.pdf]

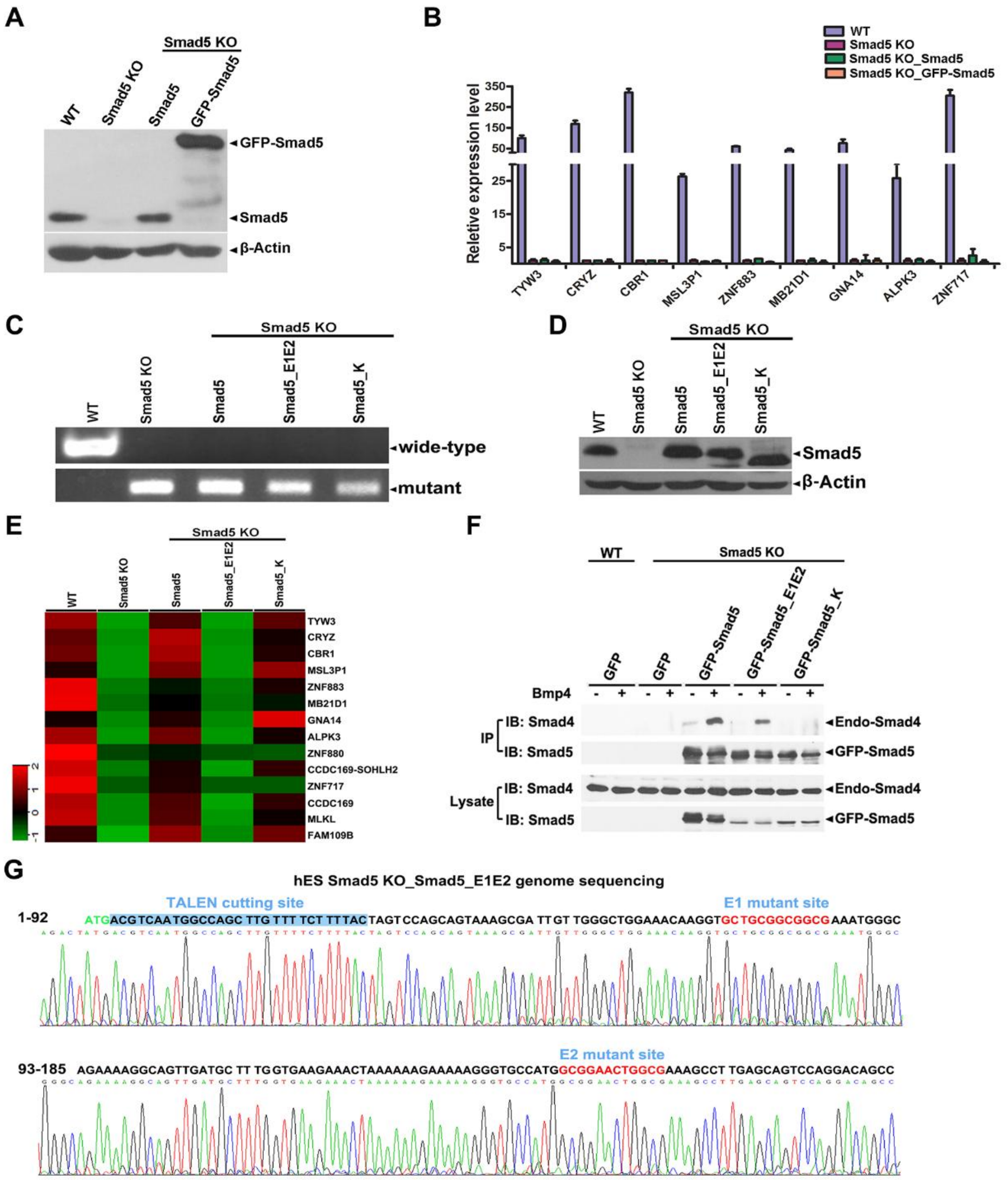
A
Smad5 KO
WT
Smad5 KO
Smad5
GFP-Smad5
GFP-Smad5
Smad5
β-Actin
B
WT
Smad5 KO
Smad5 KO_Smad5
Smad5 KO_GFP-Smad5
Reletive expression level
TYW3
CRYZ
CBR1
MSL3P1
ZNF883
MB21D1
GNA14
ALPK3
ZNF717
C
Smad5 KO
WT
Smad5 KO
Smad5
Smad5_E1E2
Smad5_K
wide-type
mutant
D
Smad5 KO
WT
Smad5 KO
Smad5
Smad5_E1E2
Smad5_K
Smad5
β-Actin
E
Smad5 KO
WT
Smad5 KO
Smad5
Smad5_E1E2
Smad5_K
TYW3
CRYZ
CBR1
MSL3P1
ZNF883
MB21D1
GNA14
ALPK3
ZNF880
CCDC169-SOHLH2
ZNF717
CCDC169
MLKL
FAM109B
F
WT
Smad5 KO
GFP
GFP
GFP-Smad5
GFP-Smad5_E1E2
GFP-Smad5_K
Bmp4
IP
IB: Smad4
IB: Smad5
Lysate
IB: Smad4
IB: Smad5
Endo-Smad4
GFP-Smad5
Endo-Smad4
GFP-Smad5
G
hES Smad5 KO_Smad5_E1E2 genome sequencing
TALEN cutting site
E1 mutant site
1-92
ATGACGTCAATGGCCAGC TTG TTT TCT TTAC TAGTCCAGCAGTAAAGCGA TTGT TGGGCTGGAAACAAGGTGCTGCGGCGGCGAAATGGGC
E2 mutant site
93-185
AGAAAAGGCAGTTGATGC TT TGGTGAAGAAACTAAAAAAGAAAAAGGGTGCCATGGCGGAACTGGCGAAAGCCT TGAGCAGTCCAGGACAGCC

Supplementary information, Figure S9. Chronic metabolic adaptation of *Smad5* KO hESCs. **(A)** Western blotting confirms the reintroducing of Smad5 or GFP-Smad5 in *Smad5* KO hESCs through lentiviral infection. **(B)** Reintroducing of Smad5 or GFP-Smad5 in *Smad5* KO hESCs fails to rescue the mRNA expression of those downregulated genes caused by *Smad5* KO. Each quantification was made from 3 independent images. Data are presented as mean ± s.e.m. **(C)** Genomic DNA PCR results showing *Smad5* KO efficiency in pre-rescued hESCs. **(D)** Western bloting showing preserved ectopic *Smad5*, *Smad5_E1E2* and *Smad5_K* expression after endogenous *Smad5* KO. **(E)** Heatmap of expression changes in WT, *Smad5* KO and pre-rescue hESCs. **(F)** HEK293 cells stably expressing *GFP*, *GFP-Smad5*, *GFP-Smad5_E1E2* or *GFP-Smad5_K* were stimulated with BMP4 for 1 hr. After immunoprecipitation with the GFP antibody, their interactions with Smad4 were detected by western blotting. **(G)** Genome sequencing of pre-rescued *Smad5_E1E2* hESCs.
