# Supplementary material for: Smad5 acts as an intracellular pH messenger and maintains bioenergetic homeostasis
Source: Cell Res. 2017 Jul 4;27(9):1083–99. doi: 10.1038/cr.2017.85 (PMC5587853; doi:10.1038/cr.2017.85)
Supplement: Supplementary information, Figure S10 — Smad5 shuttling could not regulate metabolism-related gene expression. [file cr201785x10.pdf]

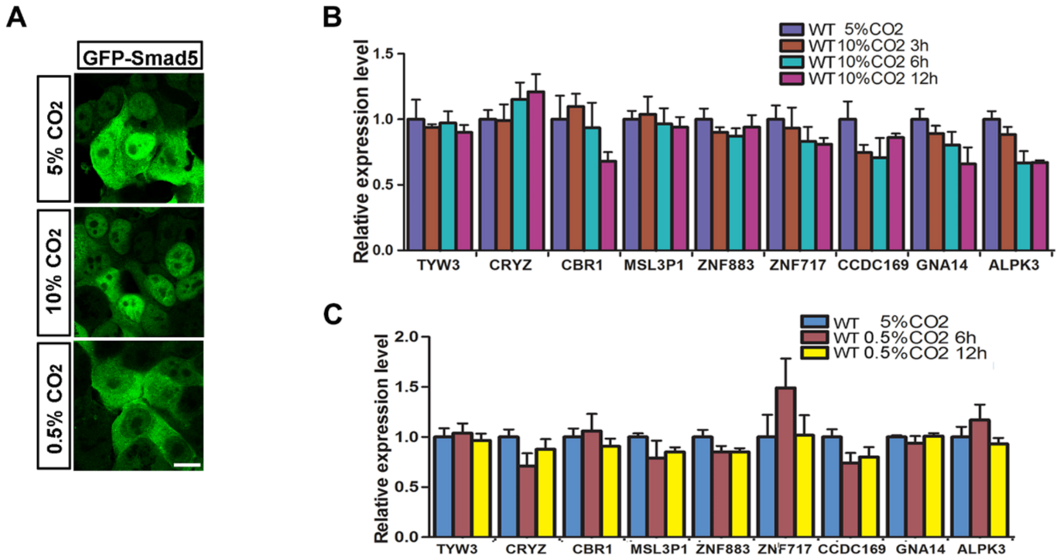
A
GFP-Smad5
5% CO2
10% CO2
0.5% CO2
B
WT 5%CO2
WT 10%CO2 3h
WT 10%CO2 6h
WT 10%CO2 12h
Relative expression level
1.5
1.0
0.5
0.0
TYW3
CRYZ
CBR1
MSL3P1
ZNF883
ZNF717
CCDC169
GNA14
ALPK3
C
WT 5%CO2
WT 0.5%CO2 6h
WT 0.5%CO2 12h
Relative expression level
2.0
1.5
1.0
0.5
0.0
TYW3
CRYZ
CBR1
MSL3P1
ZNF883
ZNF717
CCDC169
GNA14
ALPK3

Supplementary information, Figure S10. Smad5 shuttling could not regulate metabolism-related gene expression. **(A)** Long-term maintenance of extracellular pH through adjusting CO_2 levels leads to efficient accumulation of GFP-Smad5 in the nucleus (10% CO_2) or the cytoplasm (0.5% CO_2). Scale bar, 10 μm. **(B, C)** qRT-PCR analyses showing no obvious gene expression change after acute extracellular pH adjustment at 5%, 10% CO_2 and 0.5% CO_2 for 3, 6 or 12 hours. Data are presented as mean ± s.e.m of three independent experiments.
