# Supplementary material for: Smad5 acts as an intracellular pH messenger and maintains bioenergetic homeostasis
Source: Cell Res. 2017 Jul 4;27(9):1083–99. doi: 10.1038/cr.2017.85 (PMC5587853; doi:10.1038/cr.2017.85)
Supplement: Supplementary information, Figure S11 — Smad5 KO reduces glycolysis. [file cr201785x11.pdf]

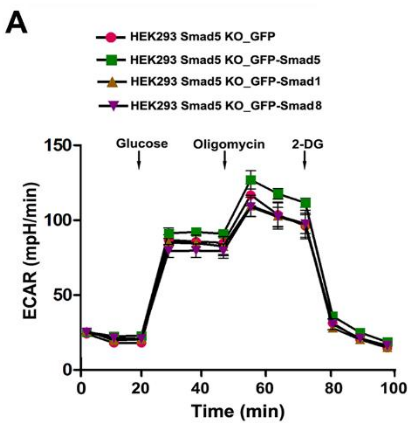
A
HEK293 Smad5 KO_GFP
HEK293 Smad5 KO_GFP-Smad5
HEK293 Smad5 KO_GFP-Smad1
HEK293 Smad5 KO_GFP-Smad8
Glucose
Oligomycin
2-DG
ECAR (mpH/min)
Time (min)
0
50
100
150
20
40
60
80

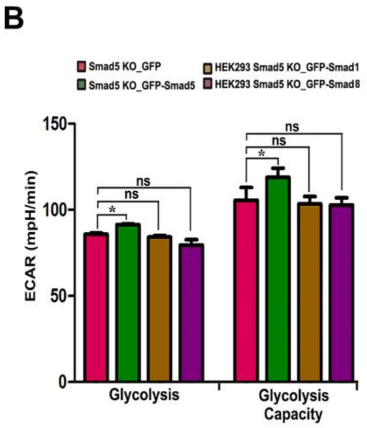
B
Smad5 KO_GFP
Smad5 KO_GFP-Smad5
HEK293 Smad5 KO_GFP-Smad1
HEK293 Smad5 KO_GFP-Smad8
ECAR (mpH/min)
0
50
100
150
ns
*
Glycolysis
Glycolysis Capacity

Supplementary information, Figure S11. *Smad5* KO reduces glycolysis. **(A)** *GFP*, *GFP-Smad5*, *GFP-Smad1* and *GFP-Smad8* were expressed in *Smad5* KO HEK293 cells, and extracellular acidification rate (ECAR) was measured by the Seahorse Analyzer (n = 6 each). **(B)** Statistics of glycolysis and glycolysis capacity in **A**. Data are represented as mean ±s.e.m of 6 independent experiments. Unpaired two-tailed Student's *t*-test. *$p < 0.05$.
