# Supplementary material for: Smad5 acts as an intracellular pH messenger and maintains bioenergetic homeostasis
Source: Cell Res. 2017 Jul 4;27(9):1083–99. doi: 10.1038/cr.2017.85 (PMC5587853; doi:10.1038/cr.2017.85)
Supplement: Supplementary information, Figure S12 — Smad5 KO induces irreversible mitochondrial morphology changes. [file cr201785x12.pdf]

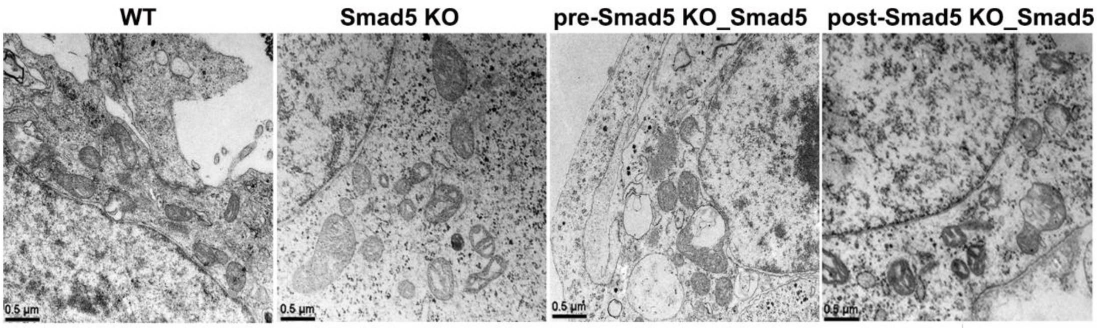
WT
Smad5 KO
pre-Smad5 KO_Smad5
post-Smad5 KO_Smad5
0.5 µm
0.5 µm
0.5 µm
0.5 µm

Supplementary information, Figure S12. *Smad5* KO induces irreversible mitochondrial morphology changes. Electron microscopy images show that re-expressing *Smad5* in *Smad5* KO hESCs through lentiviral infection could not rescue mitochondrial morphology caused by *Smad5* KO. While mitochondrial morphology could be rescued by pre-expression of *Smad5*. Scale bar, 0.5 μm.
