# Supplementary material for: Smad5 acts as an intracellular pH messenger and maintains bioenergetic homeostasis
Source: Cell Res. 2017 Jul 4;27(9):1083–99. doi: 10.1038/cr.2017.85 (PMC5587853; doi:10.1038/cr.2017.85)
Supplement: Supplementary information, Figure S13 — Smad5 KO impaired mitochondrial respiration. [file cr201785x13.pdf]

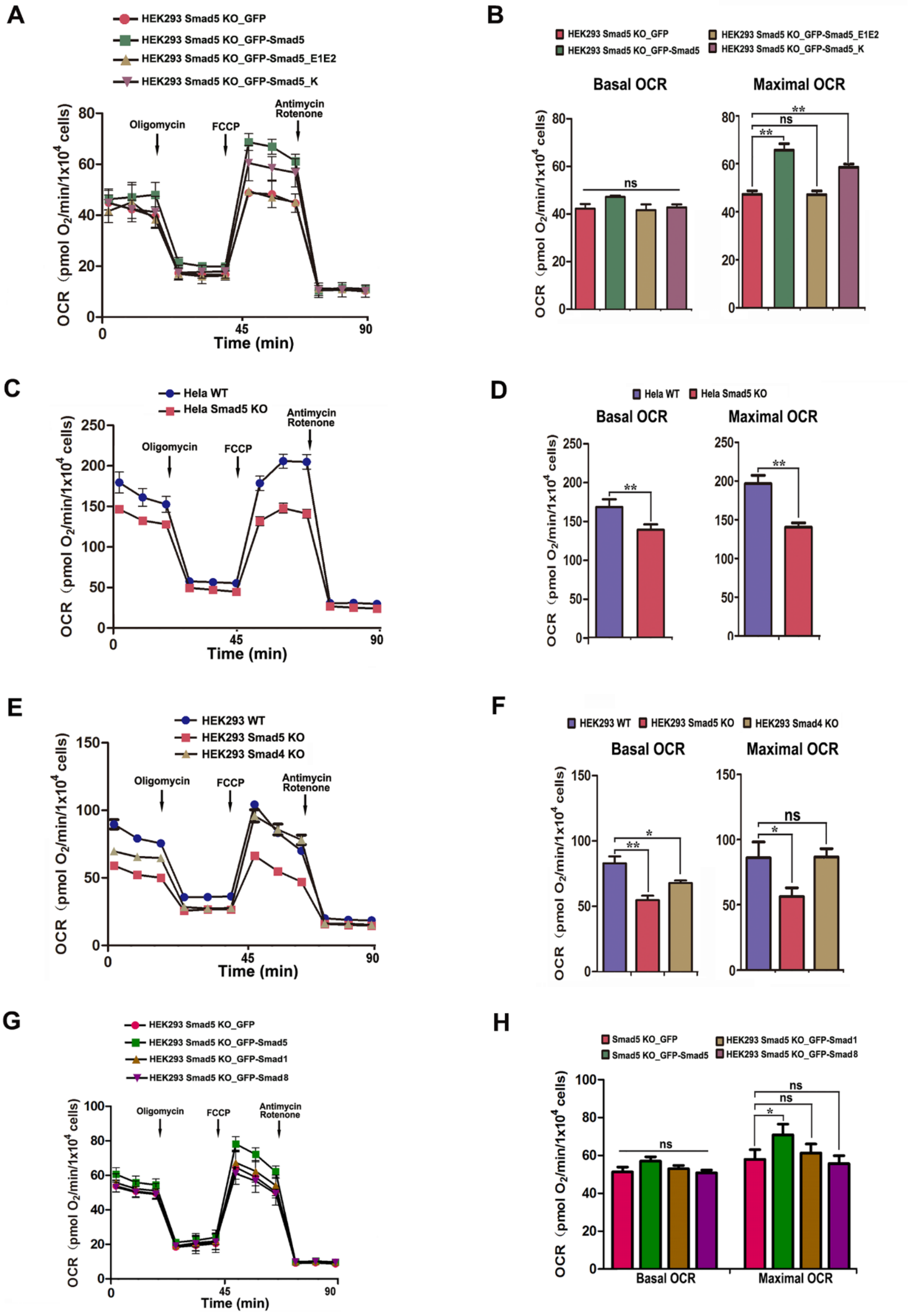
A
HEK293 Smad5 KO_GFP
HEK293 Smad5 KO_GFP-Smad5
HEK293 Smad5 KO_GFP-Smad5_E1E2
HEK293 Smad5 KO_GFP-Smad5_K
Oligomycin
FCCP
Antimycin Rotenone
OCR (pmol O2/min/1x10^4 cells)
Time (min)
B
HEK293 Smad5 KO_GFP
HEK293 Smad5 KO_GFP-Smad5
HEK293 Smad5 KO_GFP-Smad5_E1E2
HEK293 Smad5 KO_GFP-Smad5_K
Basal OCR
Maximal OCR
ns
**
C
Hela WT
Hela Smad5 KO
Oligomycin
FCCP
Antimycin Rotenone
OCR (pmol O2/min/1x10^4 cells)
Time (min)
D
Hela WT
Hela Smad5 KO
Basal OCR
Maximal OCR
**
E
HEK293 WT
HEK293 Smad5 KO
HEK293 Smad4 KO
Oligomycin
FCCP
Antimycin Rotenone
OCR (pmol O2/min/1x10^4 cells)
Time (min)
F
HEK293 WT
HEK293 Smad5 KO
HEK293 Smad4 KO
Basal OCR
Maximal OCR
**
*
ns
G
HEK293 Smad5 KO_GFP
HEK293 Smad5 KO_GFP-Smad5
HEK293 Smad5 KO_GFP-Smad1
HEK293 Smad5 KO_GFP-Smad8
Oligomycin
FCCP
Antimycin Rotenone
OCR (pmol O2/min/1x10^4 cells)
Time (min)
H
Smad5 KO_GFP
Smad5 KO_GFP-Smad5
HEK293 Smad5 KO_GFP-Smad1
HEK293 Smad5 KO_GFP-Smad8
OCR (pmol O2/min/1x10^4 cells)
Basal OCR
Maximal OCR
ns
*

Supplementary information, Figure S13. *Smad5* KO impaired mitochondrial respiration. **(A)** *GFP*, *GFP-Smad5*, *GFP-Smad5_E1E2* and *GFP-Smad5_K* were expressed in *Smad5* KO HEK293 cells, and OCR was measured by the Seahorse Analyzer (n = 6 each). **(B)** Statistics of basal and maximal OCR in **A**. Data are represented as mean ± s.e.m of 6 independent experiments. Unpaired two-tailed Student's *t*-test. **p < 0.01. **(C)** Oxygen consumption rate (OCR) changes were measured in HeLa WT and *Smad5* KO cells by Seahorse Analyzer (n = 6 each). **(D)** Statistics of basal and maximal OCR in **C**. Data are represented as mean ± s.e.m of 6 independent experiments. Unpaired two-tailed Student's *t*-test. **p < 0.01. **(E)** Oxygen consumption rate (OCR) changes were measured in HEK293 WT , *Smad5* KO and *Smad4* KO cells by Seahorse Analyzer (n = 6 each). **(F)** Statistics of basal and maximal OCR in **E**. Data are represented as mean ±s.e.m of 6 independent experiments. Unpaired two-tailed Student's *t*-test. *p < 0.05. **p < 0.01. **(G)** *GFP*, *GFP-Smad5*, *GFP-Smad1* and *GFP-Smad8* were expressed in *Smad5* KO HEK293 cells, and OCR was measured by the Seahorse Analyzer (n = 6 each). **(H)** Statistics of basal and maximal OCR in **G**. Data are represented as mean ±s.e.m of 6 independent experiments. Unpaired two-tailed Student's *t*-test. *p < 0.05.
