# Supplementary material for: Smad5 acts as an intracellular pH messenger and maintains bioenergetic homeostasis
Source: Cell Res. 2017 Jul 4;27(9):1083–99. doi: 10.1038/cr.2017.85 (PMC5587853; doi:10.1038/cr.2017.85)
Supplement: Supplementary information, Figure S14 — Increased pHi during neural differentiation. [file cr201785x14.pdf]

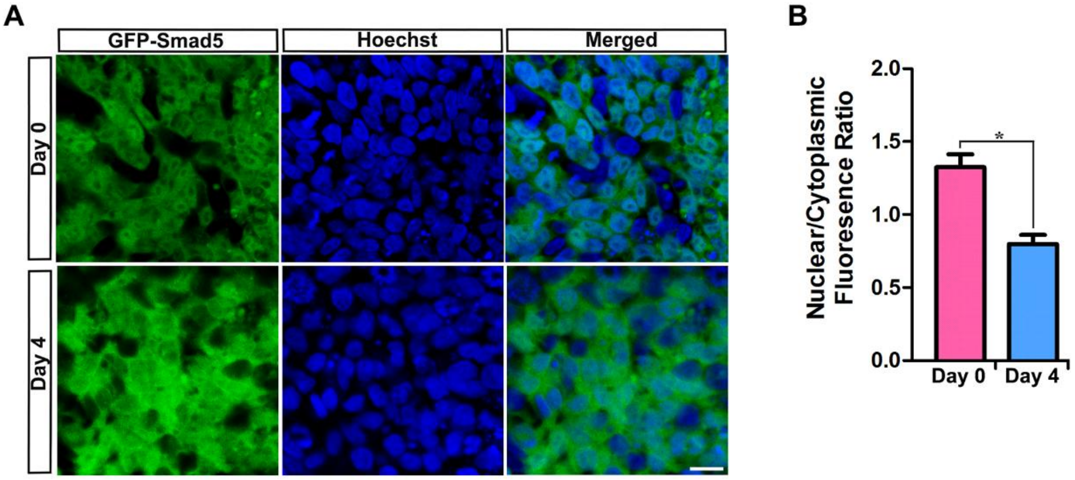
A
GFP-Smad5
Hoechst
Merged
Day 0
Day 4
B
Nuclear/Cytoplasmic Fluoresence Ratio
2.0
1.5
1.0
0.5
0.0
*
Day 0
Day 4

Supplementary information, Figure S14. Increased pHi during neural differentiation. **(A)** Distribution of GFP-Smad5 at distinct steps of neural induction in *GFP-Smad5* hESCs. Day 0, hESC stage; Day 4, neuroectoderm. Scale bar, 10 μm. **(B)** Average fluorescence quantification of nuclear and cytoplasmic localized GFP-Smad5 at different stages. (n=30; data are mean ± s.e.m. *p < 0.05).
