# Supplementary material for: Smad5 acts as an intracellular pH messenger and maintains bioenergetic homeostasis
Source: Cell Res. 2017 Jul 4;27(9):1083–99. doi: 10.1038/cr.2017.85 (PMC5587853; doi:10.1038/cr.2017.85)
Supplement: Supplementary information, Figure S15 — Smad5 interacts with HK1. [file cr201785x15.pdf]

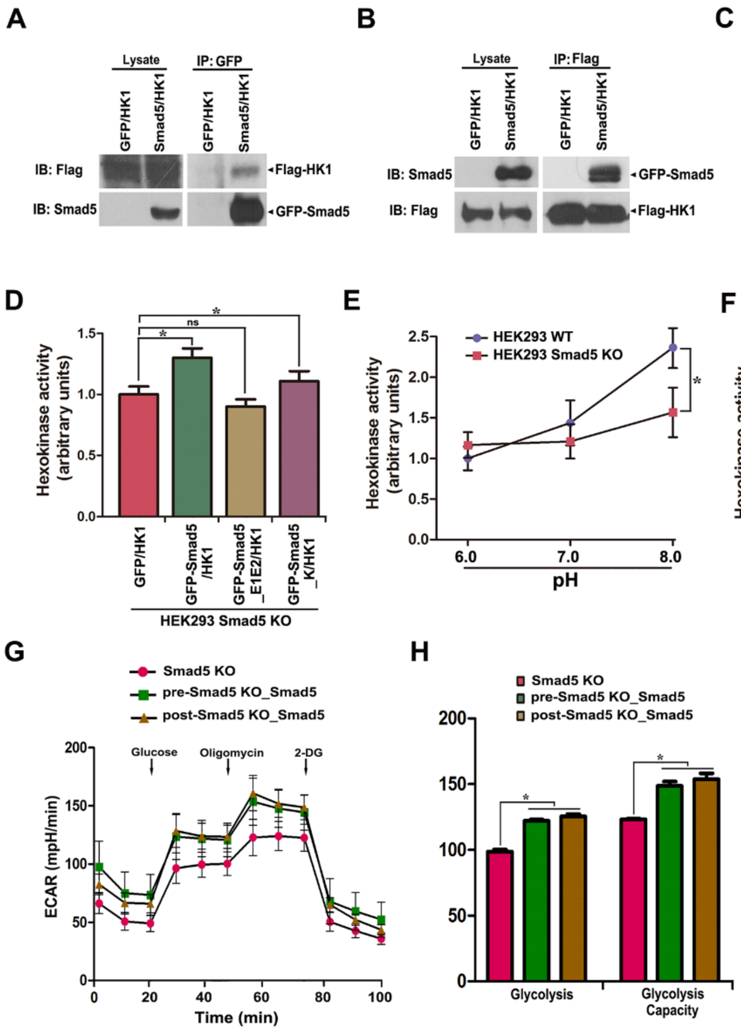
A
Lysate
IP: GFP
GFP/HK1
Smad5/HK1
IB: Flag
IB: Smad5
Flag-HK1
GFP-Smad5
B
Lysate
IP: Flag
IB: Smad5
IB: Flag
GFP-Smad5
Flag-HK1
C
hES
HEK293
Hela
Hexokinase activity (arbitrary units)
WT
Smad5 KO
D
Hexokinase activity (arbitrary units)
GFP/HK1
GFP-Smad5 /HK1
GFP-Smad5 _E1E2/HK1
GFP-Smad5 _K/HK1
HEK293 Smad5 KO
ns
E
HEK293 WT
HEK293 Smad5 KO
pH
F
Hela WT
Hela Smad5 KO
pH
G
Smad5 KO
pre-Smad5 KO_Smad5
post-Smad5 KO_Smad5
Glucose
Oligomycin
2-DG
ECAR (mpH/min)
Time (min)
H
Smad5 KO
pre-Smad5 KO_Smad5
post-Smad5 KO_Smad5
Glycolysis
Glycolysis Capacity

**Supplementary information, Figure S15.** Smad5 interacts with HK1. **(A, B)** HEK293 cells co-expressing of *Flag-HK1* and *GFP* or *GFP-Smad5* were immunoprecipitated with anti-GFP **(A)** or anti-Flag **(B)** antibody, and specific interaction of GFP-Smad5 with Flag-HK1 is shown by western blotting. **(C)** Hexokinase enzyme activity was measured in cytosolic fractions of WT and *Smad5* KO hES, HEK293 and HeLa cells. **(D)** Ectopic expressing of *GFP-Smad5* and *GFP-Smad5_K* potentiates hexokinase enzyme activity in HEK293 cells. Data are presented as mean ± s.e.m of three independent experiments. $*p < 0.05$. **(E-F)** WT and *Smad5* KO HEK293 cells **(E)** and HeLa cells **(F)** were incubated in buffers at a pH of 6.0, 7.0 or 8.0 buffer for 30 minutes, followed by hexokinase enzyme activity detection. Data are presented as mean ±s.e.m of three independent experiments. $*p < 0.05$. **(G)** Extracellular acidification rate (ECAR) measured in hESCs was rescued by either post-knockout re-expression or pre-knockout overexpression of *Smad5* (n = 6 each). **(H)** Statistics of glycolysis and glycolysis capacity in **G**. Data are represented as mean ±s.e.m of 6 independent experiments. Unpaired two-tailed Student's t-test. $*p < 0.05$.
