# Supplementary material for: Smad5 acts as an intracellular pH messenger and maintains bioenergetic homeostasis
Source: Cell Res. 2017 Jul 4;27(9):1083–99. doi: 10.1038/cr.2017.85 (PMC5587853; doi:10.1038/cr.2017.85)
Supplement: Supplementary information, Table S1 — Smad5 (cytosolic) pull down proteins [file cr201785x21.pdf]

Supplementary information, Table S1.

Smad5 (cytosolic) pull down proteins	
Identified Proteins	Gene Name
Mothers against decapentaplegic homolog 5	SMAD5
Protein piccolo	PCLO
Tubulin beta chain	TUBB
Tubulin beta-2A chain	TUBB2A
Tubulin beta-2B chain	TUBB2B
Tubulin beta-4B chain	TUBB4B
Tubulin beta-4A chain	TUBB4A
Tubulin beta-3 chain	TUBB3
ATP synthase subunit beta, mitochondrial	ATP5B
ATP synthase subunit alpha, mitochondrial	ATP5A1
Actin, alpha cardiac muscle 1	ACTC1
Sodium/potassium-transporting ATPase subunit alpha-3	ATP1A3
Dynamin-like 120 kDa protein, mitochondrial	OPA1
Heat shock protein HSP 90-beta	HSP90AB1
Myelin basic protein	MBP
Putative keratin-87 protein	KRT87P
Dihydropyrimidinase-related protein 2	DPYSL2
Sodium/potassium-transporting ATPase subunit alpha-2	ATP1A2
Heat shock protein HSP 90-alpha	HSP90AA1
Pyruvate kinase PKM	PKM
Elongation factor 1-alpha 1	EEF1A1
Protein-L-isoaspartate(D-aspartate) O-methyltransferase	PCMT1
Peroxiredoxin-1 (Fragment)	PRDX1
ADP/ATP translocase 3	SLC25A6
Glyceraldehyde-3-phosphate dehydrogenase	GAPDH
Excitatory amino acid transporter 2	SLC1A2
Enolase	ENO2
Alpha-enolase	ENO1
Cytosol aminopeptidase	LAP3
Myelin proteolipid protein	PLP1
Complement C4-B	C4B
Hexokinase-1	HK1
Histone H4	HIST1H4A
Mothers against decapentaplegic homolog 9	SMAD9
Ras-related protein Rab-1B	RAB1B
Ras-related protein Rab-10	RAB10
Clathrin heavy chain	CLTC
ATPase family AAA domain-containing protein 3A (Fragment)	ATAD3A
Guanine nucleotide-binding protein G(i) subunit alpha-1	GNAI1

Syntaxin-binding protein 1	STXBP1
Ras-related protein Rab-3A	RAB3A
Heterogeneous nuclear ribonucleoprotein H	HNRNPH1
GTP-binding nuclear protein Ran	RAN
Fructose-bisphosphate aldolase (Fragment)	ALDOA
Antithrombin-III	SERPINC1
Histone H2B type 1-K	HIST1H2BK
Histidine-rich glycoprotein	HRG
Peroxiredoxin-2	PRDX2
Alpha-internexin	INA
Neurofilament medium polypeptide	NEFM
Neurofilament light polypeptide	NEFL
Eukaryotic initiation factor 4A-II	EIF4A2
Malate dehydrogenase	MDH2
T-complex protein 1 subunit zeta	CCT6A
Synaptotagmin I, isoform CRA_b	SYT1
Guanine nucleotide-binding protein G(I)/G(S)/G(T) subunit beta-1 (Fragment)	GNB1
Calmodulin	CALM2
Calcium-transporting ATPase	ATP2B1
Prohibitin-2	PHB2
Spectrin alpha chain, non-erythrocytic 1	SPTAN1
Probable ATP-dependent RNA helicase DDX5	DDX5
Synapsin-1	SYN1
V-type proton ATPase catalytic subunit A	ATP6V1A
Peroxiredoxin-4 (Fragment)	PRDX4
Heterogeneous nuclear ribonucleoprotein M	HNRNPM
Transforming acidic coiled-coil-containing protein 3	TACC3
Cilia- and flagella-associated protein 44 (Fragment)	CFAP44
Tubulin alpha-4A chain	TUBA4A
